# Primate-specific oestrogen-responsive long non-coding RNAs regulate proliferation and viability of human breast cancer cells

**DOI:** 10.1098/rsob.150262

**Published:** 2016-12-21

**Authors:** Chin-Yo Lin, Erica L. Kleinbrink, Fabien Dachet, Juan Cai, Donghong Ju, Amanda Goldstone, Emily J. Wood, Ka Liu, Hui Jia, Anton-Scott Goustin, Mary A. Kosir, Pattaraporn Thepsuwan, Leonard Lipovich

**Affiliations:** 1 Center for Nuclear Receptors and Cell Signaling, Department of Biology and Biochemistry, University of Houston, Houston, TX 77004, USA; 2 Department of Neurology, School of Medicine, Wayne State University, Detroit, MI 48201, USA; 3 Center for Molecular Medicine and Genetics, Wayne State University, Detroit, MI 48201, USA; 4 Department of Surgery and Karmanos Cancer Institute, School of Medicine, Wayne State University, Detroit, MI 48201, USA; 5 Department of Neurology and Rehabilitation, College of Medicine, University of Illinois at Chicago, Chicago, IL 60612, USA

**Keywords:** oestrogen, long non-coding RNA, MAP kinase, evolution, cancer

## Abstract

Long non-coding RNAs (lncRNAs) are transcripts of a recently discovered class of genes which do not code for proteins. LncRNA genes are approximately as numerous as protein-coding genes in the human genome. However, comparatively little remains known about lncRNA functions. We globally interrogated changes in the lncRNA transcriptome of oestrogen receptor positive human breast cancer cells following treatment with oestrogen, and identified 127 oestrogen-responsive lncRNAs. Consistent with the emerging evidence that most human lncRNA genes lack homologues outside of primates, our evolutionary analysis revealed primate-specific lncRNAs downstream of oestrogen signalling. We demonstrate, using multiple functional assays to probe gain- and loss-of-function phenotypes in two oestrogen receptor positive human breast cancer cell lines, that two primate-specific oestrogen-responsive lncRNAs identified in this study (the oestrogen-repressed lncRNA BC041455, which reduces cell viability, and the oestrogen-induced lncRNA CR593775, which increases cell viability) exert previously unrecognized functions in cell proliferation and growth factor signalling pathways. The results suggest that oestrogen-responsive lncRNAs are capable of altering the proliferation and viability of human breast cancer cells. No effects on cellular phenotypes were associated with control transfections. As heretofore unappreciated components of key signalling pathways in cancers, including the MAP kinase pathway, lncRNAs hence represent a novel mechanism of action for oestrogen effects on cellular proliferation and viability phenotypes. This finding warrants further investigation in basic and translational studies of breast and potentially other types of cancers, has broad relevance to lncRNAs in other nuclear hormone receptor pathways, and should facilitate exploiting and targeting these cell viability modulating lncRNAs in post-genomic therapeutics.

## 1. Background

### 1.1. Human long non-coding RNA genes are abundant and multifunctional, including in cancer

Although nearly all of the genome is transcribed into various RNA molecules, protein-coding exons account for less than 2% of human genome sequence. There are approximately 20 000 protein-coding genes in the human genome. Beyond their mRNAs, which constitute the best-known part of the transcriptome, a variable number of genes transcribed into non-coding RNAs (ncRNAs) comprise more than half the transcripts in the cell [1,2]. The GENCODE catalogue of human genes, produced as part of the Encyclopaedia of DNA Elements (ENCODE) Consortium effort (the official successor to the Human Genome Project), revealed that ncRNA genes comprise the majority of the 60 000 human genes, and long non-coding (lnc) RNA genes comprise the largest ncRNA gene class [3,4]. LncRNAs are computationally defined as transcripts that harbour an open reading frame (ORF) smaller than 100 codons and devoid of known protein or domain homologies. LncRNA ORFs are poorly conserved [3]. Similar to the mRNA molecules that are the transcripts of protein-coding genes, numerous lncRNAs are transcribed by RNA polymerase II (Pol II). Moreover, many lncRNAs are capped, polyadenylated and spliced, as are mRNAs. These properties of lncRNAs facilitate high-throughput lncRNA discovery from full-length cDNA databases constructed to catalogue mammalian transcriptomes, as cDNA cloning in the underlying libraries is performed using a dual 5′-cap-trapping and 3′-end oligo-dT priming strategy [1]. Phylogenetic analysis suggests that genic species-specific and evolutionary lineage-specific sequence differences among organisms are more prevalent in ncRNA than in protein-coding genes. In the human genome, many lncRNA genes have arisen during evolution relatively recently (less than 65 Myr ago), whereas the majority of protein-coding genes display evident pan-mammalian conservation. In fact, as many as 60–80% of human lncRNA genes are not conserved beyond the primate lineage [4–6]. It has been experimentally documented that despite their low overall conservation, lncRNAs perform essential and important cellular functions in normal tissues and disease, such as contributing to nuclear architecture and serving as direct riboregulators of homeobox proteins, nuclear hormone receptors and other transcription factors [7,8]. They also modulate promoter activity and perform numerous additional functions, including sense–antisense regulation, where an antisense lncRNA regulates a protein-coding sense mRNA encoded at the same locus [8]. LncRNA genes are an order of magnitude more numerous than microRNA genes, and in contrast to microRNAs' post-transcriptional negative regulation of coding targets, lncRNAs have very diverse roles. The versatility of positive and negative, epigenetic and post-transcriptional, *cis* and *trans*, sequence-specific and global lncRNA regulatory modalities, combined with the large numbers of lncRNA genes in mammalian genomes, warrants a systematic investigation of how the regulatory language of the lncRNAome may impact normal cellular functions and disease.

A recent survey of lncRNA expression in more than 5000 tumours across 13 different cancer types from The Cancer Genome Atlas suggests that the lncRNA transcriptome is more cancer type-specific than the coding (mRNA) transcriptome [9]. Conceivably, these type-specific lncRNAs might provide the cancer cells with additional information beyond known oncogenic and tumour suppressor protein factors that drive the neoplastic process. For instance, the lncRNA metastasis-associated lung adenocarcinoma transcript 1 (MALAT1) plays a role in the metastatic potential of not only lung adenocarcinomas, but also hepatocellular carcinoma, colonic cancer and bladder cancer. The proposed mechanisms of MALAT1 action include the regulation of nuclear-splicing events in a global fashion [10] and through promoting the epithelial-to-mesenchymal transition in bladder cancers [11]. MALAT1 is merely one of a large and rapidly expanding class of examples highlighting specific lncRNA roles in cancer [12].

### 1.2. Oestrogen is mitogenic in oestrogen receptor positive breast carcinomas and can up- and downregulate protein-coding and lncRNA target genes

The role of oestrogen in breast cancer has been recognized for more than a century, since Beatson [13] demonstrated that bilateral oophorectomy resulted in the remission of breast cancer in premenopausal women. The majority of invasive breast cancers harbour functional receptors for oestrogen, including two oestrogen receptors, α and β (ERα and ERβ), members of the nuclear hormone receptor superfamily of transcription factors. When liganded to the ER, oestrogen provides a growth-stimulatory signal to the tumour that can be pharmacologically blocked by selective oestrogen receptor modulators such as tamoxifen and raloxifene. ER-positive breast cancer cell lines (such as MCF-7 and T-47D used in this study) have proven valuable for understanding the cell and molecular biology of the oestrogen response in these common breast cancers. The chief circulating oestrogen, 17β-oestradiol (E2), is lipophilic and thus readily passes through the cell's plasma membrane, where it can engage receptors first in the cytosol, and then in the nucleus where hormone-liganded ER dimers can bind in sequence-specific fashion to DNA via their DNA-binding domain. Hormone-liganded ER dimers then act as transcription factors to activate and/or repress gene transcription. The first global studies of ER-dependent transcriptional regulation focused on protein-coding genes [14–16]. Over the past several years, oestrogen-regulated ncRNA genes have begun to be elucidated [17,18].

In this study, we first built custom microarrays to interrogate transcription of more than 6000 lncRNA genes that we had catalogued from full-length GenBank cDNA sequences [19]. Upon initial identification of 127 oestrogen-responsive lncRNA genes in ERα-positive human MCF-7 cells, we selected a subset of 25 for further validation, and further narrowed this subset through multiple phenotypic assays to one pair of lncRNAs for loss-of-function and gain-of-function studies: one oestrogen-repressed lncRNA gene (BC041455) and one oestrogen-induced lncRNA gene (CR593775). We show that these two primate-specific oestrogen-regulated lncRNAs exert strong and reproducible effects on cell viability, cell growth and cell death, demonstrating this through both ablation and overexpression of both lncRNAs in two independent cell lines with two distinct phenotypic assays.

## 2. Results

### 2.1. Custom-microarray identification of 127 oestrogen-responsive lncRNAs

**Figure 1.**
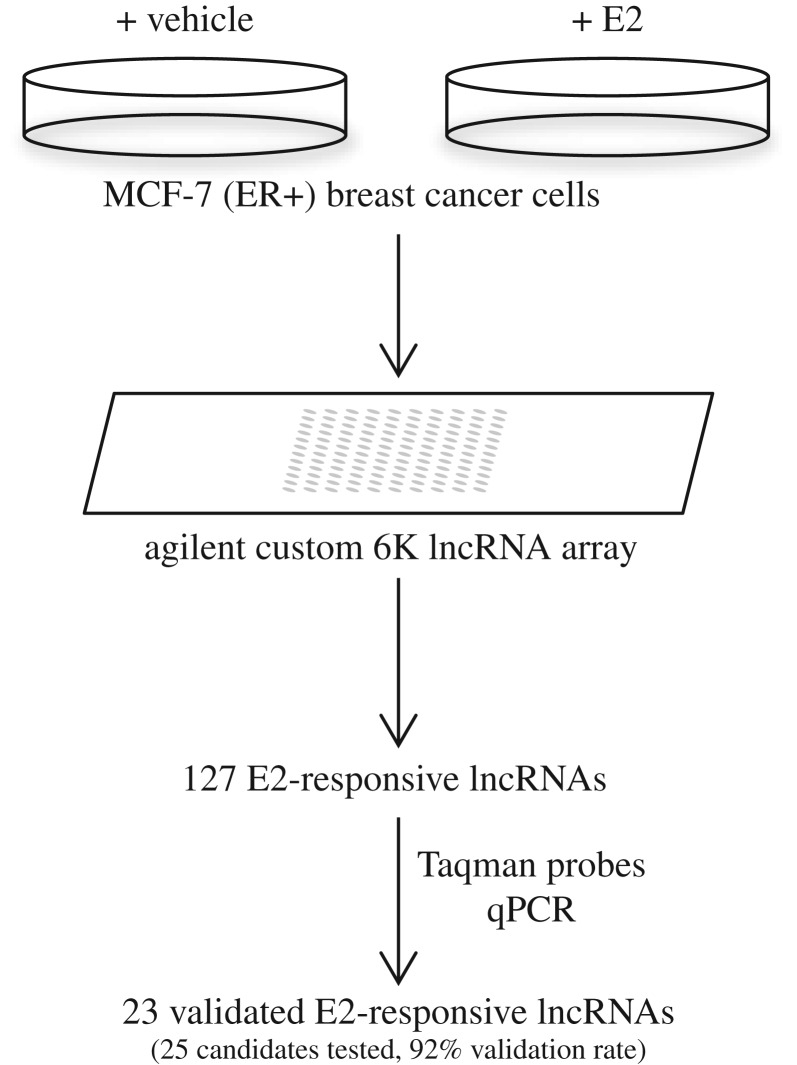
Summary and overall workflow of microarray analysis and PCR validation of oestrogen-responsive lncRNAs.

To identify oestrogen-regulated lncRNAs in breast cancer cells, we treated ERα-positive MCF-7 cells with E2 or with vehicle, and measured lncRNA expression using our custom microarrays, which detect transcripts from more than 6000 lncRNA genes that had been catalogued from full-length GenBank cDNA sequences without any conservation-based filtering [19]. Using three complementary cut-offs, a total of 127 lncRNA genes were defined as differentially expressed (DE) following E2 treatment (electronic supplementary material, table S1). Of these, 44 were upregulated by hormone treatment and 83 were downregulated. The greater number of E2-repressed lncRNA genes is consistent with observations made in previous expression studies for protein-coding genes [14]. Although there are fewer upregulated lncRNAs, their magnitude of change in response to E2 tended to be greater than for downregulated lncRNAs. There was no bias in the distribution of the DE lncRNAs across the genome. From these 127 DE lncRNAs, we selected the top 25 most significant genes for further validation and analysis (electronic supplementary material, table S2). For 23 out of 25 (92%) of the lncRNAs tested, quantitative real-time PCR (qRTPCR) on cDNA templates confirmed the microarray results (electronic supplementary material, tables S2 and S3). Validations were performed using catalogue as well as custom TaqMan^®^ primers/probe combinations purchased from Life Technologies, Inc. (electronic supplementary material, table S4). Across the 25 lncRNAs, the Pearson's correlation coefficient between the duplicate microarray and the TaqMan qRTPCR data, using the magnitude of expression level change, was +0.65 (correlation *p* < 10^−3^), suggesting that the PCR validation was generally successful. The Pearson's correlation coefficient between microarrays and qRTPCR for the 23 validated genes was +0.74 (correlation *p* < 10^−4^). The results of the microarray analysis and validation studies are summarized in figure 1.

### 2.2. Oestrogen-responsive lncRNA genes harbour ERα and FOXA1 transcription factor binding sites

For the oestrogen-responsive lncRNAs from our microarray study, we hypothesized that some are direct targets of the major oestrogen receptor, the oestrogen receptor alpha (ERα). To identify putative target genes, we assessed the presence of ERα binding sites at each lncRNA locus (5 kb upstream and 5 kb downstream of the gene body) by two complementary methods: empirical experimental binding site mapping from the ENCODE Consortium chromatin immunoprecipitation sequencing (ChIP-seq) datasets, and binding site predictions using the Dragon ERE computational tool [20]. Seven validated E2-responsive lncRNAs are adjacent to ChIP-seq mapped ERα binding sites, including six upregulated lncRNAs. One of these, CR593775, has a ChIP-seq mapped ERα binding site at its promoter (electronic supplementary material, figure S13). Three of these lncRNA gene loci (AK090603, BC041455 and CR593775) also contain ChIP-seq binding sites for FOXA1, a key cofactor required for transcriptional activation by ERα [16]. This combination of ERα and FOXA1 sites adds evidence for direct regulation of these lncRNAs by ERα. For 15 of the validated E2-responsive lncRNAs, there is no experimental evidence of ERα binding in their proximity, but computational analysis by the Dragon ERE software suggests possible binding sites within these gene loci. Only three of the top 25 DE lncRNAs have neither ChIP-seq nor Dragon ERE evidence supporting their direct regulation by ERα.

### 2.3. Human oestradiol-responsive lncRNA genes have recent evolutionary origins

LncRNA genes are less conserved than protein-coding genes at the primary sequence level [4], and well over half of human lncRNA genes are primate-specific [4–6]. Recent studies of multispecies lncRNA conservation [5,6] concluded that 60–80% of human lncRNAs are primate-specific, inspiring the idea of searching for functional, including disease-contributing, lncRNAs in the large subset of primate-specific human lncRNAs. We therefore hypothesized that some E2-responsive lncRNAs may not be conserved across mammalian lineages. We analysed key features of genomic structure that define where gene boundaries are located (consensus polyadenylation signals) and how the gene is spliced (splice sites). Of the top 25 DE (E2-responsive) lncRNA genes, 14 were spliced, and four of these lncRNA genes (CR593775, X15675, BC038366 and BC041455) had at least one primate-specific splice site (i.e. the splice donor or the splice acceptor of an intron). Canonical polyadenylation signals were identified in 14 of the top 25 DE lncRNAs, and four of these (AK057709, BC038557, BC039678 and BC041455) had primate-specific polyA signals. The wider set of 127 E2-responsive lncRNAs from the microarray mirrored these trends: from our UCSC Genome Browser-assisted manual annotation of multispecies sequence alignments for all 127 lncRNAs (electronic supplementary material, tables S2 and S5), we found that 27% [21] of canonical polyadenylation signals in the canonically polyadenylated subset (*n* = 86) of these 127 lncRNAs are primate-specific, and that 49% [22] of the canonically spliced subset of these lncRNAs (*n* = 43) harbour at least one primate-specific splice site.

AK057709 has been previously reported as an oestrogen-regulated lncRNA [17], but was not functionally pursued by the team that had first reported it. Oestrogen is a proliferative hormone and hence oestrogen-induced genes are expected to increase cell viability and proliferation. The UCSC Genome Browser shows this oestrogen-induced lncRNA to be a host of multiple piRNAs, and we found its knockdown to paradoxically increase rather than to decrease cell viability and hence decided not to pursue it as well (data not shown). The other two leads from Jonsson *et al*. [17] were LINC01016 (not in our set of 127) and LINC00160, which lacked full-length cDNA support in GenBank at the time of our study design and thus was not represented on our custom microarray. To more globally assess the relationship between our E2-responsive lncRNAs and those from recent studies of ncRNA transcriptome oestrogen responses, we performed a genomic-coordinates, strand-specific, exon-specific intersection of our 127 MCF-7 E2-responsive DE lncRNAs with the much larger catalogue of 1623 lncRNAs identified in human breast cancer subtypes as influencing overall patient survival [18]. We located 50 of our 127 genes in their dataset (electronic supplementary material, file S6). Among these 50 genes were our top E2-induced (CR593775) and top E2-repressed (BC041455) lncRNAs. The significant overlap suggests that many of the oestrogen-responsive hits from our cell-line-based screen are not cell line artefacts and may be relevant to the clinical reality of breast cancer.

The polyA signal and both splice sites of our top oestrogen-repressed lncRNA, BC041455, were primate-specific (electronic supplementary material, figure S13). Further examples of primate specificity include the lncRNA CR593775, which has a splice donor conserved only in primates and the treeshrew (the non-primate mammal phylogenetically closest to primates), and a splice acceptor conserved only in primates, except for the prosimians. Exon 2 of this lncRNA is therefore reliant on a key gene structure element that arose recently in primate evolution, in the common ancestor of Old World and New World monkeys, after the prosimian split. We defined ‘key gene structure elements’ of a gene as the canonical polyadenylation signal (AATAAA or ATTAAA) plus all canonical splice donors (GT-) and splice acceptors (-AG) of that gene, and we used the MultiZ track of the UCSC Genome Browser on the hg19 human genome assembly to interrogate the conservation of key gene structure elements for oestrogen-responsive lncRNA genes. A set-wide annotation of splice site and polyadenylation signal conservation of our oestrogen-responsive lncRNAs identified 42 lncRNAs with primate-specific gene structure elements. These included 20 lncRNAs, BC041455 among them, with completely primate-specific gene structures (electronic supplementary material, table S5). The evolutionary history of these lncRNAs implies limitations of non-primate model organisms, particularly in endogenous-gene loss-of-function studies, and mirrors our earlier finding that only 22–24% of experimentally confirmed human ERα binding sites are conserved [22].

### 2.4. An oestrogen-induced lncRNA is a positive regulator of breast cancer cell proliferation, and an oestrogen-repressed lncRNA is a negative regulator of cell viability

**Figure 2.**
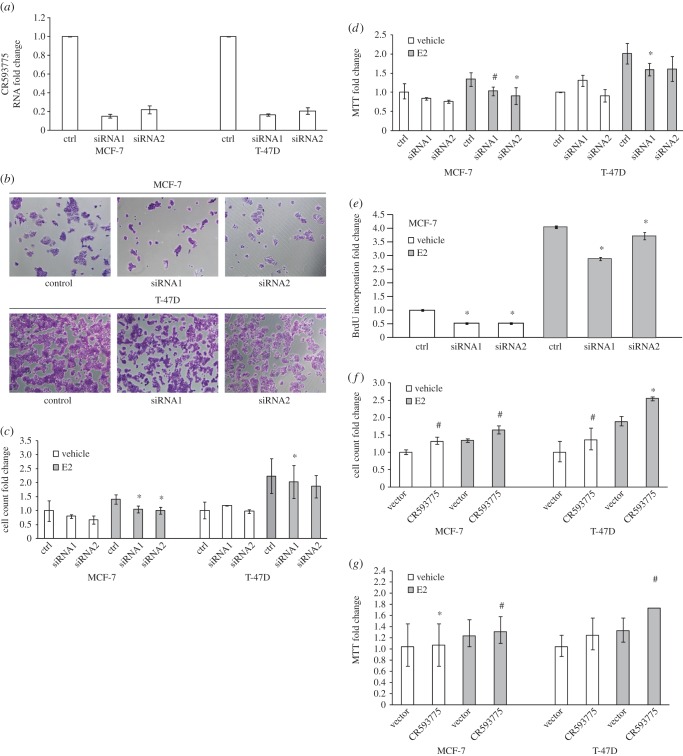
Knockdown of the oestrogen-induced lncRNA CR593775 inhibits breast cancer cell proliferation and viability, whereas overexpression of the same lncRNA enhances breast cancer cell proliferation and viability. (*a*) Validation of siRNA-mediated silencing (‘knockdown’) of CR593775 expression was performed by TaqMan qRTPCR. (*b*) Images of MCF-7 and T-47D cells following control (scrambled) siRNA or targeting siRNA1 or siRNA2 (two different sequences) show decreases in cell number following knockdown. Only E2-treated cells were used in (*b*). (*c*) Quantification of the effects of CR593775 knockdown in both MCF-7 and T-47D cells on cell numbers, as assessed by crystal violet staining followed by cell counting. (*d*) Effects of both siRNAs were confirmed by tetrazolium salt (MTT) reduction assays. (*e*) SiRNA-mediated knockdown of CR593775 decreased DNA synthesis in breast cancer cells, as shown by BrdU incorporation assays. (*f*) Overexpression of CR593775 increased cell proliferation, as determined by crystal violet staining followed by cell counting. (*g*) Increased cell proliferation was also detected by MTT assays in cells overexpressing CR593775. Three biological replicates were included in each experiment, with the exception of the BrdU studies that achieved statistical significance with two replicates. For all graphs, asterisks denote statistically significant differences between control and knockdown or overexpression experiments as determined by Student's *t*-test (*p* < 0.05). Hashes denote near significant differences (0.05 < *p* < 0.01). Fold-change values are relative to the E2– control treatment with either vector or control siRNA. Fold-change values for the E2– control treatment with vector or control siRNA have been set to 1 but the error bars reflect variations between each biological replicate to the mean of all replicates. Error bars represent standard errors of the mean. We use white bars and grey bars to distinguish no-E2 and E2-treated cells, respectively (*c*–*g*).

Because oestrogen is a positive regulator of cell growth, we posited that E2-responsive lncRNAs may be involved in hormone-dependent growth and proliferation of breast cancer cells. Moreover, we hypothesized that lncRNAs upregulated by E2 treatment are likely to be positive regulators of cell proliferation, whereas lncRNAs downregulated by E2 may exert inhibitory effects on proliferation. To test this hypothesis, we first disrupted the expression of the E2-upregulated lncRNA CR593775 by RNA interference. ENCODE Consortium stranded LongRNAseq subcellular compartment transcriptome tracks in the UCSC Genome Browser (data not shown) revealed appreciable cytoplasmic localization of CR593775, indicating that it should be amenable to small interfering RNA (siRNA)-mediated knockdowns, properties shared by other lncRNAs in our prior work in mammalian systems [21,23]. Knockdown of CR593775 expression by two different siRNAs designed to target CR593775 was successfully validated by TaqMan qRTPCR (figure 2*a*), and significantly reduced the number of viable breast cancer cells when compared with the scrambled siRNA-transfected controls (figure 2*a*,*b*). As expected, treatment with E2 increased cell proliferation. The effects of our two different CR593775 knockdown siRNAs—which reduced cell proliferation—were observed in both vehicle- and E2-treated cells in the ER-positive breast cancer cell line MCF-7 (although they were observed only after E2 treatment in T-47D; figure 2*c*). Furthermore, these results (a reduction in cell viability upon CR593775 knockdown) were confirmed by tetrazolium salt reduction MTT assays (figure 2*d*). To determine whether disruption of CR593775 affects DNA synthesis, a hallmark of cell proliferation, bromodeoxyuridine (BrdU) incorporation assays were carried out in control and targeting siRNA-transfected MCF-7 cells (figure 2*e*). The proportion of cells which stained positive for BrdU was lower in the siRNA-transfected cells when compared with the controls, and this effect was more pronounced under oestrogen stimulation. These results suggest that CR593775 is involved in mediating the downstream effects of oestrogen signalling on breast cancer cell proliferation, including possible interactions with mechanisms of DNA synthesis regulation. Cell cycle analysis in synchronized cells indicates that disruption of CR593775 expression did not affect cell cycle progression (data not shown).

**Figure 3.**
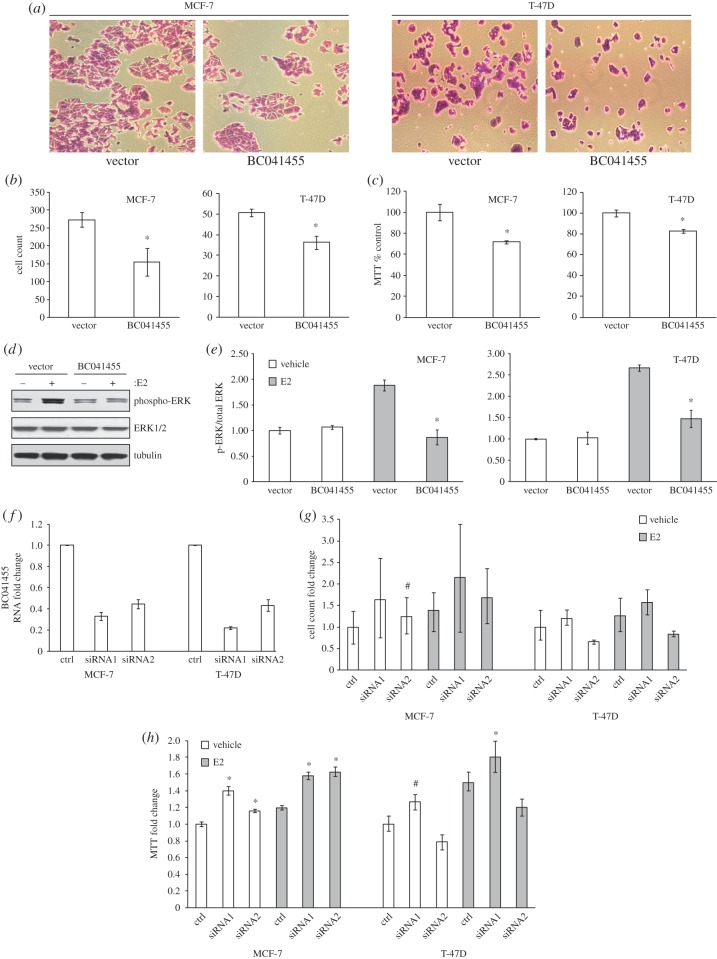
Overexpression of the oestrogen-repressed lncRNA BC041455 inhibits breast cancer cell proliferation and viability as well as a growth factor signalling pathway, while knockdown of the same lncRNA enhances breast cancer cell proliferation and viability. (*a*) Crystal violet staining of MCF-7 cells shows reduced numbers of cells following overexpression of BC041455. (*b*) Quantification of the effects of BC041455 overexpression in both MCF-7 and T-47D cells, using crystal violet cell count. (*c*) Effects of BC041455 overexpression were confirmed by tetrazolium salt (MTT) reduction assays. (*d*) Overexpression of BC041455 decreased ERK phosphorylation. (*e*) Quantification of ERK phosphorylation using densitometry of bands confirms a decrease following BC041455 overexpression. (*f*) Knockdown of BC041455 expression by two siRNA sequences (siRNA1 and siRNA2) was validated by TaqMan qRTPCR. (*g*) siRNA-mediated knockdown of BC041455 expression increased cell proliferation, as determined by crystal violet staining and cell count in MCF-7 cells (while there was no consistent effect in T-47D cells). (*h*) MTT assay of cell viability and proliferation showed similar trends. Three biological replicates are included in each experiment and error bars represent standard errors of the mean. Fold-change values for the E2– control treatment with vector or control siRNA have been set to 1, but the error bars reflect variations between each biological replicate to the mean of all replicates. Asterisks denote statistically significant differences (*p* < 0.05 using Student's *t*-test) between vector and overexpression or scramble siRNA and targeting siRNA experiments. Hashes denote near significant differences (0.05 < *p* < 0.01). In (*h*), we compared the MTT assay results of experiments with each BC041455-knockdown siRNA, independently, to control siRNA, without E2 (*h*; left MCF-7 and right T-47D panels, left side of each panel, white bars). Separately, we compared the values of each siRNA, independently, to control siRNA, in cells treated with E2 (*h*; left MCF-7 and right T-47D panels, right side of each panel, grey bars). We use white bars and grey bars to distinguish no-E2 and E2-treated cells, respectively (*e*,*g*,*h*).

We then performed functional studies of BC041455, a primate-specific lncRNA downregulated by E2 treatment. BC041455 exhibits a chiefly cytoplasmic localization in the cell (electronic supplementary material, document S7). This pattern, seen previously for other lncRNAs, is classified by Cabili *et al*. [24] as type V, where puncta representing lncRNAs are scattered throughout the cytoplasm. The mainly cytoplasmic localization of BC041455 is also supported by ENCODE Consortium LongRNAseq subcellular compartment tracks in the UCSC Genome Browser (electronic supplementary material, document S12). The promoter of BC041455 is endogenously bound in multiple human cell types by CEBPβ (electronic supplementary material, figure S13), a transcription factor that is a known inducer of cell death in breast cancer [25]. CEBPβ binds directly at the transcription start site of BC041455 in nearly all human cell lines which have available ENCODE Consortium ChIP-seq data. This indicates that BC041455 may be a contributing effector of CEBPβ-induced cell death, as a direct target of this transcription factor. Overexpression of BC041455 reduced the number of viable cells (figure 3*a*) in both MCF-7 and T-47D ER-positive cell lines compared with the empty vector controls (figure 3*b*). This reduction in cell proliferation was also reflected in MTT assays in these cells (figure 3*c*). We examined the effects of overexpressing BC041455 on the activity of the mitogen-activated protein kinase (MAPK) signalling cascade, a growth regulatory signalling pathway known to be activated by E2 [26,27]. In the absence of E2, expression of BC041455 did not affect the phosphorylation of the MAPKs extracellular signal-regulated kinase (ERK)1/2. Following E2 treatment, overexpression of BC041455 prevented ERK1/2 phosphorylation (figure 3*d*). The effects of BC041455 on hormone-dependent MAPK signalling were observed in both MCF-7 and T-47D cells (figure 3*e*). We conclude that BC041455 may function as a ‘brake’ in growth factor signalling, where it may exert tumour-suppressive roles.

### 2.5. Overexpression and knockdown of E2-repressed and E2-induced lncRNAs affect cell proliferation and viability

To functionally reverse-complement our initial loss-of-function assays of the E2-repressed lncRNA BC041455 (overexpression) and E2-induced lncRNA CR593775 (knockdown), we performed the reciprocal, gain-of-function assays by knocking down BC041455 and overexpressing CR593775. We hypothesized that these gain-of-function experiments would result in enhanced cell proliferation and increased cell viability testable by the same phenotypic assays (MTT assay; crystal violet staining with both automated and manual cell counting; see Material and methods). After starvation for both natural oestrogens (switching to charcoal-stripped fetal bovine serum (FBS)) and xenoestrogens (switching to phenol-red-free DMEM), MCF-7 and T-47D cells consistently responded mitogenically to 10 nM E2. Furthermore, the results support the prediction of enhanced cellular proliferation with both BC041455 siRNA knockdown and CR593775 overexpression (figures 2*f*,*g* and 3*f*–*h*). CR593775 overexpression enhanced cell proliferation in MCF-7 cells, as measured by crystal violet cell count (effect size approx. 31% in E2– cells and approx. 22% after E2+ induction, *p*-value < 0.001; figure 2*f*). In T-47D, CR593775 overexpression effects in the E2– background were weak and variable, consistent with no effect (although a non-significant increase in cell proliferation was observed), but after E2+ induction a consistent 30% effect was apparent (*p*-value 0.0024; figure 2*f*). BC041455 siRNA knockdown enhanced cell proliferation in MCF-7 cells, both with and without oestrogen. Effects were strong and significant in the E2– background (effect size approx. 50% and approx. twofold, *p*-values 0.007 and 0.006 for the two siRNAs, respectively), and somewhat weaker after E2+ induction (effect size approx. 50% and approx. 70%, *p*-values 0.047 and 0.03; figure 3*g*). Taken together, these observations strongly support the trend of enhanced proliferation (Fisher's method *p*-value 3.5 × 10^−4^). Notably, these BC041455-knockdown siRNAs lacked consistent effects in T-47D cells (figure 3*f–h*), underscoring the potential importance of the differences in genetic background and mutational landscape of these two cell lines. These phenotypes were broadly concordant in MTT assays from the same transfection experiments (figure 2*g*,*h* and electronic supplementary material, document S8) and were reproducible in three biological replicates with deep (fourfold) technical replication. All lncRNA knockdown and overexpression experiments were successfully validated by TaqMan qRTPCR (figures 2*a* and 3*f* and electronic supplementary material, document S9). Summarily, the reproducibility of phenotypes at the opposite ends of the cell viability and proliferation spectrum upon knockdown and overexpression of CR593775 and BC041455 point to these lncRNAs as a proliferation driver and a proliferation brake, respectively, in human breast cancer cells.

## 3. Discussion

Our study of the oestrogen-responsive lncRNA transcriptome in breast cancer cells identified 127 lncRNAs whose expression levels were regulated by oestrogen, representing 2% of the non-coding transcriptome interrogated by the array. This is close to the percentage (3%) of oestrogen-responsive mRNAs [14,28]. Similar to mRNAs, the majority of DE lncRNAs were downregulated by E2 treatment. The top-ranked lncRNAs, based on significance and magnitude of differential expression, tend to be upregulated by oestrogen and include some with experimentally mapped ERα and FOXA1 binding sites in their *cis*-regulatory regions, indicative of their direct regulation by this nuclear hormone receptor.

Several lines of experimental evidence point to direct regulation of the characterized lncRNAs by factors involved in ER and breast cancer biology. Promoter regions of both genes contain empirically mapped (ENCODE ChIP-seq) binding sites for ERα and FOXA1. ERα plays a pivotal role in regulating gene expression in breast cancer cells in response to the binding of oestrogen. FOXA1 serves a pioneering factor in facilitating transcriptional activation of ER target genes [29]. Its expression in breast tumours is associated with hormone-responsive cancers and good prognosis. In cancer cells, oestrogen drives cell proliferation and survival, and ER signalling is a major prognostic marker and therapeutic target. The binding of CEBPβ, a transcription factor shown to regulate cancer cell survival, to the promoter of BC041455 further suggests clinical significance of the target lncRNAs. Their hormone-responsive status, along with their effects on cellular proliferation and ERK phosphorylation, indicates involvement in growth factor signalling pathways of central clinical importance in breast cancer. Experimentally determined histone modification marks indicate that these gene loci reside in open chromatin and are likely to be under the regulation of complex transcription factor regulatory networks (electronic supplementary material, figure S13*c*,*d*). Taken together, these results suggest that these lncRNAs may function in early regulatory events of oestrogen signalling, possibly through interactions with key proteins in the signalling cascades and checkpoints which drive proliferation of breast cancer cells.

Both oestrogen-independent and oestrogen-dependent regulation may take place at the BC041455 and CR593775 lncRNA loci. Specifically, histone marks and chromatin immunoprecipitation experiment results at the BC041455 locus (ENCODE ChIP-seq, UCSC Genome Browser; electronic supplementary material, figure S13) are consistent with regulation through a variety of transcription factor binding sites in many cell types. Sixty-nine of the 125 cell types profiled by ENCODE have a DNase1 hypersensitive site (DHS1 site) at the BC041455 promoter, indicating diverse and complex regulation of this lncRNA in numerous human tissue types, and in primary (non-cancer) cells.

Similar to the sole intron of BC041455, ENCODE ChIP-seq analysis of transcription factors and histone modifications indicates that the sole intron of CR593775 contains a biochemically hyperactive region, with epigenetic signatures broadly consistent with enhancer activity [2]. An analogous signature occurs at the CR593775 promoter, indicating that the lncRNA CR593775 may be an enhancer RNA (eRNA) [30,31]. These sites are in single-copy sequence, not in repeats, supporting their biological validity. Several oncogenic transcription factors with roles not limited to nuclear hormone receptor pathways, including MYC, bind the CR593775 promoter and intron, consistent with the proliferative function of this gene that we demonstrated, and implying potential oestrogen-dependent as well as oestrogen-independent regulation of cell proliferation. There is minimal evolutionary conservation at these sites in the MultiZ 100-species alignment, consistent with non-conservation of the gene structure beyond primates (electronic supplementary material, S13*c*,*d*), and hence the phenotypes we observed are consistent with biological function in the absence of deep evolutionary conservation. The diversity and complexity of promoter and intronic regulation of CR593775 points to a possible plurality of functional roles, including non-oestrogen-dependent modalities. This may explain our observation of cell death upon CR593775 knockdown in the triple-negative breast cancer cell line MDA-MB-231, which lacks the oestrogen receptor (electronic supplementary material, document S10).

The CR593775 and BC041455 loci contain enhancer of Zeste homologue 2 (EZH2) binding sites. EZH2 is a component of the Polycomb repressor complex, involved in lncRNA-mediated silencing of protein-coding target genes. Expression rescue of epigenetically lncRNA-silenced genes is becoming a potential goal of targeted therapeutics [32]. Known Polycomb-partnering regulatory RNAs include the rapidly evolving lncRNA HOTAIR [33]. Future studies of oestrogen-responsive lncRNAs with cell-proliferation phenotypes should examine the interplay between their evolutionary conservation and their potential as both targets and effectors of epigenetic regulators.

Our chief result presented here is that two lncRNAs (CR593775 and BC041455) regulate cell life and death *in vitro*. Our finding that lncRNAs with potential roles in cancer pathogenesis, including CR593775 and BC041455, may be unique to primate genomes points to the limitations of non-primate disease models for drug discovery. This contrasts to drug-targeting approaches taken for genes with known roles in carcinogenesis such as MYC, RAS and HER2/neu, which are conserved among mammals, and protein coding. Although some primate-specific cancer lncRNAs may be amenable to mouse xenograft models, they cannot be studied *in vivo* in non-primates in endogenous-gene knockout contexts. If additional primate-specific lncRNAs relevant to neoplastic regulation are discovered in the future, the field may have to embrace the notion that certain mechanisms of human carcinogenesis cannot be properly modelled in rodents. On the other hand, the evolutionary uniqueness of these lncRNAs may make them attractive as therapeutic targets, bypassing the potential adverse or toxic effects of targeting protein-coding genes in conserved pathways that involve many additional network partners. Our functional studies of two representative lncRNAs (CR593775 and BC041455) indicate that oestrogen-induced and oestrogen-repressed lncRNAs can function as positive and negative, respectively, regulators of cell viability and proliferation. Cancer- and tissue-specific expression of lncRNAs may synergize with these functions, rendering these lncRNAs worthy of consideration as therapeutic targets [12]. The mechanisms of action for oestrogen-responsive, including non-conserved, lncRNAs and their expression and function in tumours remain to be determined, but represent a novel significant aspect of oestrogen signalling and breast cancer biology warranting further investigation in basic and translational studies.

## 4. Material and methods

### 4.1. Microarray analysis of oestrogen-responsive lncRNAs

To provide E2-starved and E2-stimulated RNA for our initial microarray analysis, MCF-7 cells were cultured in Dulbecco's modified Eagle's medium (DMEM, Fisher, USA) supplemented with 1% penicillin and 10% FBS at 37°C in 5% CO_2_. Cells were starved of oestrogen in 5% charcoal-stripped FBS in phenol-red-free DMEM for 72 h and treated with 10 nM 17β-oestradiol (E2, Sigma-Aldrich, USA). Control cells were treated with only the ethanol vehicle. After 24 h of treatment, cells were harvested and total RNA was extracted, using an RNAeasy Mini kit following the manufacturer's protocols (Qiagen, Valencia, CA). The first-strand cDNA was prepared, using a SuperScript III First-Strand cDNA kit according to the manufacturer's instructions (Invitrogen, USA) and analysed using custom microarrays, designed with the Agilent Technologies OpenGenomics eArray interface and seven strand-specific non-repetitive 60-mer probes per lncRNA gene as previously described in [19]. The arrays contain probes for 5586 lncRNAs. Target cDNAs were labelled with Alexa 647 and Alexa 555 in two independent processes. Following hybridization, the arrays were scanned with an Agilent Technologies Microarray Scanner. Array and images were analysed using Agilent Feature Extraction software (v. 10.3.1) with the default protocol (GE2_107_Sep09). Data were processed using the R statistical software package. To increase the accuracy of fold-change calculations, spike-in control probes from Agilent and TaqMan qRTPCR results from six control mRNAs were used to identify a slight correction of the microarray signal by applying an exponent of 1.125. Global *q*-values were computed using ANOVA results, a linear-mixed model, false discovery rate (FDR) correction and the library ‘fdrtool’. DE genes were defined using the following criteria: (i) an absolute-value fold change greater than or equal to 1.4, (ii) FDR less than or equal to 5%, (iii) all seven probes of the gene having the same direction of regulation (both up- or downregulated) as well as all seven being significant by the two preceding criteria. TaqMan qRTPCR was performed using the 7500 Fast Real-Time PCR system (Applied Biosystems, Life Technologies, USA).

### 4.2. Manual annotation of lncRNA gene structure and evolutionary conservation

We defined all canonical GT–AG splice sites (where at least one cDNA or EST of each lncRNA gene was spliced) and all canonical (AATAAA, ATTAAA) polyadenylation signals as key gene structure elements. We localized key gene structure elements by manual annotation of cDNA/EST-to-genome alignments of specific lncRNA gene transcripts in the UCSC Genome Browser [34] (Kent *et al.* [34]; genome.ucsc.edu; accessed at various times between 13 April 2015 and 23 May 2016). All non-singleton transcript isoforms, i.e. those supported by more than one cDNA or EST, were analysed for each gene. To determine evolutionary conservation, we configured the MultiZ alignment track to display in ‘full’ mode, and set the display of all vertebrate species' genomes in the alignment to ‘on’ using the UCSC advanced track configuration functionality. We then zoomed in to less than 100 bp (the maximum genomic interval size for displaying base-by-base sequence and text rather than for graphical alignment in the UCSC Genome Browser) centred on each splice site and polyadenylation signal. We canvassed the available non-human primate and non-primate genomes to determine whether each key gene structure element of the human lncRNA gene was present or absent in each such non-human genome, and, if present, whether the consensus (GT–AG, AATAAA, ATTAAA) was conserved. We reviewed each lncRNA gene's structure relative to any overlapping and nearby (within 10 kb) known protein-coding genes. We surveyed genomic positional relationships (proximity and overlap, if any) of each lncRNA gene with any overlapping and nearby known genes in a strand-specific fashion.

### 4.3. ERα binding site localization

We searched for ERα binding sites at the transcription start site (promoter), in the gene body, and within 5 kb upstream and downstream of the gene in the UCSC Genome Browser. We displayed the ENCODE TFBS track in full mode for ERα ChIP-seq (Hudson-Alpha Institute; track ‘Transcription factor binding sites by ChIP-seq from ENCODE/HAIB’) for T-47D cells. (ERα MCF-7 datasets were not available at the UCSC ENCODE portal at the time of our query.) For genes without ERα ChIP-seq hits, we used the Dragon ERE Finder software [20] to predict ERα binding sites with default parameters and repeat-masked (www.repeatmasker.org) sequence of the same genomic interval as input. At loci where empirical (ChIP-seq) and/or predicted (Dragon ERE) ERα binding sites existed, we visually searched in the UCSC Browser for ChIP-seq evidence of other transcription factor binding sites relevant to nuclear receptor signalling, including FOXA1, the main cofactor of ERα [29].

### 4.4. SiRNA-mediated knockdown of oestrogen-responsive lncRNAs

Manual annotation of each lncRNA gene in the UCSC Genome browser was performed to identify specific lncRNA targets and then siRNA sequences were designed using the Dharmacon webtool (GE Life Sciences). Cells were plated in DMEM containing 10% FBS and, after overnight culture, cells were transfected with 100 nM siRNA, using DharmaFECT 1 transfection reagent (GE Dharmacon; Lafayette, CO) according to the manufacturer's instructions. Cells transfected with the scrambled control siRNA were used as controls. After an incubation of 48 h post-transfection at 37°C, RNA was extracted, using the RNeasy kit (Qiagen, Valencia, CA), and TaqMan qRT PCR quantification was performed to check siRNA knockdown efficiency. The knockdown was defined as successful when siRNA reduced target gene expression by at least 40%. The complete results of TaqMan knockdown validation for both BC041455 and CR593775, in biological triplicates, with two siRNAs (of different sequence) per lncRNA, in both MCF-7 and T-47D cells, and including siRNA sequences, are presented in figures 2*a* and 3*f*, and electronic supplementary material, table S3.

### 4.5. Analysis of cell proliferation using crystal violet staining and MTT reduction assays

For crystal violet staining, cells were fixed with 4% paraformaldehyde diluted into PBS from a 16% solution purchased from EM Sciences (catalogue 15710; Hatfield, PA). The cells were stained with 0.05% crystal violet (product 12785; EM Sciences) dissolved in PBS, washed three times with distilled water then allowed to dry at room temperature before photographing. Stained cells were imaged with the 10× objective (Nikon Japan V.25 160 Ph1 DL) and four fields per well were photographed in RGB colour, using a SPOT RT3 CCD camera (SPOT Imaging, Sterling Heights, MI).

To automatically quantify the cell counts, .jpg2 or .tif images from the microscope-attached camera were imported into the automated image analysis software Cell Profiler v. 2.1.1 (Broad Institute, Cambridge, MA). All images were imported into the input module as follows: first is ‘images’, on the module setting specify with images only. Then select ‘no’ on metadata. For name and type, on the module setting assign name to ‘all images’, select the image type to greyscale image, name to assign to the images name as raw, and click the update button at the bottom of the module setting to upload all images assigned. The analysis module on Cell Profiler v. 2.1.1 was set with five different modules. The first module was Image Math, to invert the resolution of regular greyscale images to a lower resolution in order to give out better count. The second module was Identify Primary Object. The options in this module were set to use the Image AfterMath by adjusting the threshold and differentiated resolution. In order to distinguish the clumps and detect the cell nuclei, we set the threshold smoothing scale and the correction factor to 1.0. The third module was Measure Object Size Shape; in this module, we changed the setting to only measuring the nuclei. The fourth module was Identify Secondary Object; we use the watershed option in this module to identify a secondary object by differentiating the nuclei that are too close to each other (this is a key aspect of successful automated cell counting, because MCF-7 and T-47D cells often ‘clump’ or reside with nuclei of multiple cells in close proximity). The last module was Export to Excel Spreadsheet; in this setting, we chose the output folder. After all the modules had been set and images had loaded, we clicked the ‘Analysing Images’ button for the program to begin the cell counting process. The data were then exported to Microsoft Excel. We provide a representative screenshot of our CellProfiler software usage (electronic supplementary material, document S11). We had extensively optimized CellProfiler settings for automated counting of individual cells prior to arriving at these settings, and we always validated all automated cell counting experiments by manually counting cells from four image areas per cell culture plate well. Subsequently, we graphed the data using Microsoft Excel 2013.

For the MTT assay, cells were plated at 5000–10 000 cells per well (96-well plate). We starved the cells to remove oestrogenic substances and then treated with 17β-oestradiol (E2) or vehicle (ethanol) for 48 h. After 48 h of E2 treatment, thiazolyl blue tetrazolium bromide (MTT; Sigma Aldrich, USA; 5 mg ml^−1^ in PBS) was incorporated into the 96-well plate for 3.5 h at 37°C. The reduced dye was solubilized using 150 µl of DMSO, with mixing on the orbital shaker for 15 min. The absorbance of converted dye was measured at 690 and 590 nm using the Molecular Dynamics VERSAmax plate reader. We subtracted the 690 nm absorbance values from the 590 nm absorbance values. Biological triplicates were performed, and we exported the data to Microsoft Excel.

### 4.6. BrdU incorporation assays

Transfected cells were pulsed with 10 µM BrdU for 1 h, trypsinized, fixed in 70% ethanol and stored at −20°C for 24 h. DNA was denatured in 2 M HCl/0.5% Triton-X and then neutralized in 100 mM sodium borate. FITC-conjugated anti-BrdU antibody was then added to bind incorporated BrdU. Fixed cells were incubated at 37°C for 30 min with 50 µg ml^−1^ of propidium iodide and 10 µg ml^−1^ RNase A. The FACS Aria 111 Cell Sorter (BD Biosciences) was used for data collection and data were analysed using the FlowJo software program.

### 4.7. Overexpression of lncRNAs

We obtained the full-length BC041455 clone from Open Biosystems (catalogue no. EHS1001-7376338). We cloned full-length CR593775 in-house. To amplify CR593775 from MCF-7 cDNA (SuperScript III), we used the following primers:

CR593775-R: CACATGGAAACCTGTAGCAGC
CR593775-F: CCAGGCTCTGGCTCACTCCCTC.

We initially cloned the PCR product into Topo TA v. 2.1 cloning vector from Invitrogen. To make sure it was the correct clone, we used *Hind*III and *Xba*1 restriction enzyme to digest TA cloning product and release the insert. We excised from the gel the correct insert and purified the DNA using the QIAquick^®^ gel extraction kit. The purified product was cloned into pcDNA 3.1(–) vector. We then used the DNA plasmid Miniprep kit from Invitrogen to prepare the plasmid DNA of CR593775 in our expression vector. After the plasmid Miniprep was done, we sent the plasmid samples for Sanger sequencing. We sequenced four clones full-length bidirectionally in order to verify insert sequence, insert completeness and insert orientation prior to overexpression.

Using restriction sites derived from the pCR2.1 plasmid, we shuttled the respective cDNAs into the expression plasmid pcDNA3.1(+) from ThermoFisher Scientific, and verified cDNA directionality using T7 and BGH primers. The primers used to sequence the CR593775 insert in the pcDNA3.1 vector were as follows:

T7 universal primer (source: GeneWiz): TAA TAC GAC TCA CTA TAG GG
BGHR (source: GeneWiz): TAG AAG GCA CAG TCG AGG
M13 forward (source: IDT Integrated DNA Technologies): GTA AAA CGA CGG CCA GT
M13 reverse (source: IDT Integrated DNA Technologies): CAG GAA ACA GCT ATG AC.

For transient transfection of plasmids bearing CR593775 cDNA, BC041455 cDNA or the empty vector (pcDNA3.1(+)), MCF-7 and T-47D cells were maintained in phenol-red-free DMEM and RPMI1640 containing 5% and 10% charcoal FBS without antibiotics, respectively. Cells were transfected with BC041455, and in separate experiments with CR593775 (both cloned into the constitutive expression vector pcDNA3.1), using the Lipofectamine 2000 reagent (Invitrogen) or the Fugene reagent (Promega). For subsequent phenotypic assays, cells were analysed with MTT and separately stained with crystal violet as described in the gene knockdown studies. We validated all overexpression using TaqMan qRTPCR in biological triplicates of both cell lines (electronic supplementary material, document S9).

### 4.8. Western blot analysis of ERK phosphorylation

Transfected cells were treated with 10 nM E2. One hour prior to harvest, cells received a second E2 treatment, and were lysed in RIPA buffer containing protease and phosphatase inhibitors. Cell lysates were then centrifuged at 10 000*g* for 20 min to remove debris. Protein concentrations were measured using a BCA protein assay kit (Pierce). Proteins separated by SDS–PAGE were transferred onto nitrocellulose membranes. After blocking with blocking buffer (Tris-buffered saline, 0.05% Tween 20, 5% dry skim milk, pH 7.4) for 1 h at room temperature, membranes were incubated for 24 h with phospho-ERK antibody at 1 : 1000 dilution (Cell Signaling Technology, Beverly, MA). After incubation with secondary antibodies conjugated with HRP, proteins were visualized using an ECL detection kit (Pierce) according to the manufacturer's instructions. The membranes were then stripped with a stripping buffer (Pierce) and re-probed with total ERK antibody (Cell Signaling no. 9102). To control for total protein loading, membranes were also probed with an anti-tubulin antibody (Abcam). We used the following catalogue numbers and vendors:

**Table:** 

catalogue number	item	source
89900	*RIPA* buffer	Thermo Scientific
o4906845001	Phosstop (phosphatase inhibitor)	Roche
p8340	protease inhibitors	Sigma
46430	stripping buffer	Thermo Scientific
9101	phospho-p44/42 MAPK(Erk1/2)	Cell Signaling
9102	p44/42 MAPK(Erk1/2)	Cell Signaling
ab59680	anti-tubulin 55kD	Abcam

### 4.9. Cell lines and propagation

MCF-7 cells used in this study were early-passage (p10) provided by the Lin Laboratory, originally established at the Michigan Cancer Foundation by Herb Soule from a pleural effusion of a malignant breast cancer in a 69-year-old American woman [35]. The T-47D cells established from a pleural effusion from a 54-year-old Israeli woman [36] were obtained from the American Type Culture Collection (Manassas, VA) at high passage (p86). Both these express oestrogen receptors and respond mitogenically to 17β-oestradiol. Before using for transfection, MCF-7 and T-47D cells were routinely checked for their ability to respond to 10 nM 17β-oestradiol using MTT assay and/or crystal violet staining. A third breast cancer cell line (MDA-MB-231) is triple-negative (no HER2/neu amplification and no receptors for progesterone or oestrogen), derived from a 51-year-old Caucasian woman seen at M. D. Anderson Cancer Center [37]. The basal medium used for MCF-7 and MD-MBA-231 was RPMI 1640; routine propagation of all lines used 10% FBS, with regular freeze-back of cells in liquid nitrogen. To deprive the ER-positive lines of all oestrogenic substances, cells were rinsed in phenol-red-free media (also used in trypsinization) and cultured in FBS that had been stripped of natural oestrogens, using charcoal–dextran as described [38]. The female fibroblast cell line GM03814 was obtained from the Coriell Institute for Medical Research (Camden, NJ).

### 4.10. Single-molecule Stellaris^®^ RNA-FISH localization of BC041455 lncRNA in cells

A collection of 14 RNA 19-mers (see electronic supplementary material, document S7) labelled with Quasar 570 was purchased from Biosearch Technologies (Petaluma, CA), designed to tile along the 1651 nt BC041455 lncRNA (also known as LOC101928233). Cells for FISH were grown at low density on collagen-coated glass coverslips, fixed in 4% paraformaldehyde (PBS), washed two time with PBS and stored at −20°C in 70% ethanol until hybridization, per the protocol provided by Cabili *et al.* [24] . The pooled collection of probes was hybridized for 16 h at 37°C in formamide-containing buffer (Biosearch Technologies, catalogue no. SMF-HB1-10), and then coverslips were washed extensively to remove unbound probe. Subsequently, hybridized coverslips were inverted onto glass slides with mounting media containing the DNA dye 4′,6-diamidino-2-phenylindole (DAPI) before photography. Coverslips were examined under an AxioObserver inverted fluorescence microscope (Carl Zeiss Microscopy, Jena, Germany) equipped with objective lens 63×/1.40 oil and with four Colibri 2 light-emitting diodes (LEDs; 365, 470, 555 and 625 nm). The 365 nm LED was used to image the DAPI fluorophore; the 555 nm LED was used to image the Quasar 570 fluorophore. Images were captured, using an AxioCam MRm camera (Carl Zeiss Microscopy, Jena, Germany). We acquired approximately 25–30 optical slices at 0.24–0.30 µm intervals, thereby covering the entire vertical extent of the cell. Brilliant red puncta denote the Quasar 570 signal from single RNA molecules; the blue-coloured DAPI staining shows cell nuclei. To estimate autofluorescence, we stimulated with the 470 nm LED; the signal in the red channel in such experiments was diffusely over the cytoplasm, unlike the distinct red puncta seen when the slide was illuminated with the correct, probe-specific 555 nm LED.

## Supplementary Material

Primate-specific oestrogen-responsive long non-coding RNAs regulate proliferation and viability of human breast cancer cells, Lipovich et al. Supplementary Table 1

## Supplementary Material

Primate-specific oestrogen-responsive long non-coding RNAs regulate proliferation and viability of human breast cancer cells, Lipovich et al. Supplementary Table 2

## Supplementary Material

Primate-specific oestrogen-responsive long non-coding RNAs regulate proliferation and viability of human breast cancer cells, Lipovich et al. Supplementary Table 3

## Supplementary Material

Primate-specific oestrogen-responsive long non-coding RNAs regulate proliferation and viability of human breast cancer cells, Lipovich et al. Supplementary Table 4

## Supplementary Material

Primate-specific oestrogen-responsive long non-coding RNAs regulate proliferation and viability of human breast cancer cells, Lipovich et al. Supplementary Table 5 (ALSO: Supp. Docs 6 - 11 follow; please see main text document for the numbers and names of all 11 ESMs)

## Supplementary Material

Supplementary Table 6

## Supplementary Material

Supplementary Figure 7

## Supplementary Material

Supplementary Figure 8

## Supplementary Material

Supplementary Figure 9

## Supplementary Material

Supplementary Figure 10

## Supplementary Material

Supplementary Figure 11

## Supplementary Material

ENCODE Gingeras

## Data accessibility

All supplementary files are available on figshare.
