# Supplementary material for: Primate-specific oestrogen-responsive long non-coding RNAs regulate proliferation and viability of human breast cancer cells
Source: Open Biol. 2016 Dec 21;6(12):150262. doi: 10.1098/rsob.150262 (PMC5204119; doi:10.1098/rsob.150262)
Supplement: Primate-specific oestrogen-responsive long non-coding RNAs regulate proliferation and viability of human breast cancer cells, Lipovich et al. Supplementary Table 1 [file rsob150262supp1.docx]

**Supplementary Table 1: List of 127 estrogen-responsive lncRNAs identified by microarray analysis of MCF7 cells**. Genes were declared differentially expressed when they meet three criteria: (i) an absolute-value fold change greater than or equal to 1.4 (this ratio expresses RNA levels in cells treated with estrogen, divided by the expression of the same RNA without stimulation by estrogen), (ii) a FDR less than or equal to 5%, (iii) all the seven probes of the gene having the same direction of regulation (both up or down regulated) as well as all seven being significant by the two preceding criteria. The differentially expressed lncRNA were ranked by computing first the median fold change and median FDR for their seven probes, then these were normalized by dividing by the mean. The averages of these values on the two replicate arrays were computed, then a weight of 20% was assigned to the normalized FDR and 80% to the normalized fold change. Finally a weighted composite score ("score," second column) was computed by subtracting the normalized FDR from the normalized fold change. Fold change and relationship with the nearest protein-coding gene are in subsequent columns (flank10000 = protein-coding gene exists within < 10 kb of either boundary of the lncRNA gene but they do not overlap).

| Rank | Score | lncRNA | FC | Type |
| --- | --- | --- | --- | --- |
|  |  |  |  |  |
| 1 | 3.53 | CR593775 | 9.17 | no overlap |
| 2 | 3.20 | AF086466 | 8.3 | flank10000 |
| 3 | 2.49 | BC038557 | 6.26 | no overlap |
| 4 | 2.06 | AK090603 | 4.9 | overlap |
| 5 | 1.96 | AK025743 | 4.86 | no overlap |
| 6 | 1.82 | AK057709 | 4.33 | no overlap |
| 7 | 1.66 | CR612213 | 4.44 | overlap |
| 8 | 1.57 | AF251187 | 4.18 | overlap |
| 9 | 1.51 | BC016787 | 3.45 | overlap |
| 10 | 1.49 | BC039678 | 3.98 | no overlap |
| 11 | 1.48 | AL833160 | 4.15 | flank10000 |
| 12 | 1.47 | BC038366 | 3.95 | overlap |
| 13 | 1.46 | BC040572 | 3.45 | flank10000 |
| 14 | 1.44 | BC036599 | ‑4.03 | no overlap |
| 15 | 1.43 | AK127565 | 3.46 | no overlap |
| 16 | 1.39 | BC041455 | ‑3.82 | no overlap |
| 17 | 1.38 | X15675 | 3.34 | flank10000 |
| 18 | 1.36 | hTF30525 | 2.91 | flank10000 |
| 19 | 1.35 | CR610499 | ‑3.60 | no overlap |
| 20 | 1.28 | BC038580 | 2.63 | no overlap |
| 21 | 1.27 | AK096780 | ‑2.83 | no overlap |
| 22 | 1.21 | AL832444 | ‑2.78 | no overlap |
| 23 | 1.2 | AK024898 | ‑2.43 | flank10000 |
| 24 | 1.2 | CR592608 | 2.86 | overlap |
| 25 | 1.18 | AK123408 | ‑2.63 | overlap |
| 26 | 1.15 | AK097377 | ‑2.21 | overlap |
| 27 | 1.14 | BC031342 | ‑2.14 | flank10000 |
| 28 | 1.12 | AK095250 | ‑2.29 | no overlap |
| 29 | 1.10 | AK095831 | ‑2.15 | overlap |
| 30 | 1.08 | CR592782 | ‑2.12 | no overlap |
| 31 | 1.07 | AK092525 | ‑2.28 | no overlap |
| 32 | 1.07 | AK125142 | 2.25 | overlap |
| 33 | 1.06 | AJ609455 | 2.04 | overlap |
| 34 | 1.06 | AK095849 | 1.98 | overlap |
| 35 | 1.05 | AK123622 | ‑2.12 | flank10000 |
| 36 | 1.02 | AK055150 | ‑2.67 | flank10000 |
| 37 | 1.02 | BC004287 | ‑1.95 | flank10000 |
| 38 | 1.01 | AF086556 | ‑2.11 | overlap |
| 39 | 1.01 | AK130095 | ‑2.78 | no overlap |
| 40 | 1.00 | hTF36235 | ‑1.99 | flank10000 |
| 41 | 1.00 | BX649128 | ‑1.93 | flank10000 |
| 42 | 0.97 | AF086547 | ‑1.85 | flank10000 |
| 43 | 0.97 | BC042048 | ‑2.53 | no overlap |
| 44 | 0.97 | AF086375 | ‑1.93 | no overlap |
| 45 | 0.96 | AL360200 | ‑1.84 | flank10000 |
| 46 | 0.95 | AL713639 | ‑2.00 | overlap |
| 47 | 0.95 | AK091337 | ‑1.84 | flank10000 |
| 48 | 0.94 | X96660 | 1.81 | overlap |
| 49 | 0.93 | AY264285 | 2.05 | overlap |
| 50 | 0.93 | AK128017 | 2.13 | overlap |
| 51 | 0.93 | AK123861 | ‑1.63 | flank10000 |
| 52 | 0.92 | CR612362 | ‑2.21 | no overlap |
| 53 | 0.92 | BC041955 | ‑1.79 | flank10000 |
| 54 | 0.91 | BC041657 | ‑2.26 | flank10000 |
| 55 | 0.91 | BC033335 | ‑2.26 | flank10000 |
| 56 | 0.91 | CR602684 | ‑1.84 | overlap |
| 57 | 0.91 | AK098100 | ‑2.07 | flank10000 |
| 58 | 0.90 | CR621005 | ‑1.97 | overlap |
| 59 | 0.90 | AL080082 | ‑2.04 | flank10000 |
| 60 | 0.90 | BC045192 | 1.86 | no overlap |
| 61 | 0.88 | AK000776 | ‑2.08 | flank10000 |
| 62 | 0.88 | BC040979 | ‑1.86 | overlap |
| 63 | 0.88 | X74862 | ‑1.64 | overlap |
| 64 | 0.87 | BC015457 | 2.01 | overlap |
| 65 | 0.87 | BC047034 | 1.74 | overlap |
| 66 | 0.86 | oncomir1 | 1.97 | overlap |
| 67 | 0.85 | BC036485 | ‑1.53 | flank10000 |
| 68 | 0.85 | CR591392 | ‑2.09 | no overlap |
| 69 | 0.85 | AF086154 | ‑2.09 | no overlap |
| 70 | 0.84 | AK096988 | 2.27 | flank10000 |
| 71 | 0.84 | BC041872 | ‑1.61 | overlap |
| 72 | 0.83 | AK056098 | ‑1.59 | overlap |
| 73 | 0.82 | AK123669 | ‑1.66 | flank10000 |
| 74 | 0.82 | BX538341 | ‑1.94 | flank10000 |
| 75 | 0.82 | BX641058 | ‑1.51 | flank10000 |
| 76 | 0.82 | AK000454 | ‑2.04 | overlap |
| 77 | 0.81 | AK057720 | ‑1.63 | overlap |
| 78 | 0.81 | AK057093 | 1.69 | overlap |
| 79 | 0.80 | BC031683 | 1.55 | flank10000 |
| 80 | 0.80 | AK094521 | ‑1.95 | flank10000 |
| 81 | 0.80 | AK095308 | ‑1.70 | no overlap |
| 82 | 0.80 | AK056376 | 1.46 | overlap |
| 83 | 0.79 | BX538340 | ‑1.98 | no overlap |
| 84 | 0.79 | BC015852 | ‑1.83 | flank10000 |
| 85 | 0.79 | BC015429 | 1.45 | no overlap |
| 86 | 0.78 | BC041400 | ‑1.56 | flank10000 |
| 87 | 0.78 | AL110176 | 1.97 | flank10000 |
| 88 | 0.78 | HAR1R | ‑1.49 | overlap |
| 89 | 0.78 | AL110290 | ‑1.63 | flank10000 |
| 90 | 0.78 | BC050302 | ‑1.53 | flank10000 |
| 91 | 0.77 | AJ000096 | ‑1.71 | overlap |
| 92 | 0.77 | AL137429 | ‑1.83 | overlap |
| 93 | 0.77 | AK054653 | ‑1.65 | flank10000 |
| 94 | 0.77 | BC071821 | 1.61 | flank10000 |
| 95 | 0.77 | AF075027 | ‑1.48 | flank10000 |
| 96 | 0.77 | X15624 | 1.52 | overlap |
| 97 | 0.76 | BC015977 | ‑1.68 | no overlap |
| 98 | 0.76 | AK096743 | ‑1.52 | flank10000 |
| 99 | 0.76 | CR601020 | ‑1.59 | flank10000 |
| 100 | 0.76 | AK097323 | ‑1.68 | overlap |
| 101 | 0.76 | AK056401 | 1.53 | overlap |
| 102 | 0.76 | BC031308 | 1.71 | flank10000 |
| 103 | 0.75 | BC033162 | ‑1.93 | flank10000 |
| 104 | 0.75 | CR624511 | ‑1.51 | flank10000 |
| 105 | 0.73 | BC033590 | ‑1.56 | overlap |
| 106 | 0.73 | AK096268 | ‑1.55 | overlap |
| 107 | 0.72 | AK023739 | ‑1.91 | overlap |
| 108 | 0.72 | BC035135 | ‑1.62 | flank10000 |
| 109 | 0.71 | AK056358 | ‑1.70 | flank10000 |
| 110 | 0.71 | CR603327 | 1.64 | overlap |
| 111 | 0.71 | AK055271 | ‑1.62 | flank10000 |
| 112 | 0.71 | AL109959 | ‑1.49 | overlap |
| 113 | 0.71 | AF075047 | ‑1.76 | overlap |
| 114 | 0.70 | CR611155 | 1.63 | flank10000 |
| 115 | 0.70 | AK125271 | ‑1.74 | overlap |
| 116 | 0.69 | CR597362 | ‑1.76 | flank10000 |
| 117 | 0.69 | AK023464 | 1.64 | no overlap |
| 118 | 0.69 | BC044257 | ‑1.63 | overlap |
| 119 | 0.68 | BC018684 | 1.71 | flank10000 |
| 120 | 0.67 | AF075057 | ‑1.86 | flank10000 |
| 121 | 0.66 | CR594811 | ‑1.63 | flank10000 |
| 122 | 0.66 | AK124307 | ‑1.44 | flank10000 |
| 123 | 0.64 | AK124746 | 1.53 | overlap |
| 124 | 0.62 | BC062470 | ‑1.58 | flank10000 |
| 125 | 0.62 | AF086143 | 1.51 | flank10000 |
| 126 | 0.62 | AL137571 | ‑1.51 | overlap |
| 127 | 0.61 | AF086431 | ‑1.44 | flank10000 |
