# Supplementary material for: Primate-specific oestrogen-responsive long non-coding RNAs regulate proliferation and viability of human breast cancer cells
Source: Open Biol. 2016 Dec 21;6(12):150262. doi: 10.1098/rsob.150262 (PMC5204119; doi:10.1098/rsob.150262)
Supplement: Primate-specific oestrogen-responsive long non-coding RNAs regulate proliferation and viability of human breast cancer cells, Lipovich et al. Supplementary Table 2 [file rsob150262supp2.docx]

**Supplementary Table 2: Expression, regulation, and evolution of 26 lncRNAs.** Estrogen-responsive fold changes were measured by microarray and validated by qRTPCR. ERE binding sites were identified using ChIPseq (experimental) and DRAGON algorithm (prediction). Bold underlined: lncRNA selected for RNAi experiments.

|  | Fold Change | |  |  | Ere α binding site | | | | |  |  | Evolution | | |
| --- | --- | --- | --- | --- | --- | --- | --- | --- | --- | --- | --- | --- | --- | --- |
| lncRNA | Microarray | qrtPCR |  | presence | | CHIPseq/Dragon | quantity | localizaton | Other TFBS | Site conserved |  | Splice sites (*) | polyA |  |
| **AF086466** | 8.36 | -1.11 |  | Predicted | | DRAGON |  |  |  |  |  | Unspliced | No PolyA |  |
| **AF251187** | 4.39 | 1.54 |  | No | | No |  |  |  |  |  | Unspliced | No PolyA |  |
| AK024898 | -2.41 | -3.16 |  | Predicted | | DRAGON |  |  |  |  |  | Unspliced | No PolyA |  |
| **AK025743** | 4.86 | 3.26 |  | Yes | | CHIPseq | 2 | exonic | C-myc | Tenrec |  | Vertebrate | Placental Mammal |  |
| **AK057709** | 5.67 | 5.39 |  | Yes | | CHIPseq | 1 | exonic | C-myc | Sloth |  | Placental Mammal | old World monkeys |  |
| AK090603 | 4.91 | 1.41 |  | Yes | | CHIPseq | 2 | intronic | ESF1, STAT3, FOXA1 | Orangutan |  | Vertebrate | Placental Mammal |  |
| AK096780 | -2.84 | -3.98 |  | Predicted | | DRAGON |  |  |  |  |  | Unspliced | Placental Mammal |  |
| AK123408 | -2.67 | -3.26 |  | Predicted | | DRAGON |  |  | C-Myc, E2F1 |  |  | Unspliced | Placental Mammal |  |
| **AK127565** | 3.66 | 1.55 |  | Predicted | | DRAGON |  |  |  |  |  | Vertebrate | Vertebrate |  |
| AL832444 | -2.81 | -3.06 |  | Predicted | | DRAGON |  |  |  |  |  | Unspliced | No PolyA |  |
| **AL833160** | 4.13 | 6.67 |  | Predicted | | DRAGON |  |  | FOXa1 |  |  | Unspliced | No PolyA |  |
| **BC016787** | 3.46 | 1.73 |  | Predicted | | DRAGON |  |  | E2F1, p300, GATA, FOXa1 |  |  | Placental Mammal | Placental Mammal |  |
| BC036599 | -3.96 | -1.9 |  | No | | No |  |  |  |  |  | Unspliced | No PolyA |  |
| **BC038366** | 4.04 | 1.34 |  | Yes | | CHIPseq | 1 | exonic | E2F1 | Baboon |  | Primate | Placental Mammal |  |
| BC038557 | 6.31 | -1.1 |  | Yes | | CHIPseq | 1 | 800 bp downstream | E2F1, GATA | Marmoset |  | Placental Mammal | Old world monkey |  |
| **BC038580** | 2.64 | 1.24 |  | Predicted | | DRAGON |  |  |  |  |  | Placental Mammal | Placental Mammal |  |
| **BC039678** | 4.41 | 2.67 |  | Predicted | | DRAGON |  |  |  |  |  | Vertebrate | Old world monkey |  |
| **BC040572** | 3.46 | 1.5 |  | No | | No |  |  | C-Myc, E2F1 |  |  | Placental Mammal | Placental Mammal |  |
| **CR592608** | 2.94 | 1.45 |  | Predicted | | DRAGON |  |  | STAT3, E2F |  |  | - | No PolyA |  |
| BC041455 | -3.71 | -4.23 |  | Yes | | CHIPseq | 2 | exonic | p300, C-Myc, Foxa1, GATA3 |  |  | Primate | Primate |  |
| **CR593775** | 9.29 | 2.87 |  | Yes | | CHIPseq | 2 | exonic | C-myc, FoxA1 | MegaBat |  | old World monkeys | Placental Mammal |  |
| CR610499 | -3.81 | -1.72 |  | Predicted | | DRAGON |  |  |  |  |  | - | No PolyA |  |
| **CR612213** | 4.64 | 1.4 |  | Predicted | | DRAGON |  |  | STAT3, p300 |  |  | Vertebrate | No PolyA |  |
| **HTF30525** | 2.93 | 1.09 |  | Predicted | | DRAGON |  |  |  |  |  | - | No PolyA |  |
|  |  |  |  |  | |  |  |  |  |  |  |  |  |  |
| **X15675** | 3.33 | 1.29 |  | Predicted | | DRAGON |  |  |  |  |  | Chimp | No PolyA |  |

ERV1: Endogeneous retroviral sequence 1, “‑“ indicate no PolyA signal in human, (*) the results correspond to the less conserved site regardless of whether it is the donor or the acceptor site. ENCODE ChIPseq peaks were manually analyzed in the UCSC Browser within a maximum 5kb distance from the gene boundaries and in breast cancer cell lines only.
