# Supplementary material for: Primate-specific oestrogen-responsive long non-coding RNAs regulate proliferation and viability of human breast cancer cells
Source: Open Biol. 2016 Dec 21;6(12):150262. doi: 10.1098/rsob.150262 (PMC5204119; doi:10.1098/rsob.150262)
Supplement: Primate-specific oestrogen-responsive long non-coding RNAs regulate proliferation and viability of human breast cancer cells, Lipovich et al. Supplementary Table 3 [file rsob150262supp3.docx]

**Supplementary Table 3: SiRNA knockdown validations and post-knockdown phenotypes after RNAi of estrogen-induced lncRNAs in MCF‑7 cells.** Two siRNAs were designed for each lncRNA. The reference gene (i.e. the standard) for all TaqMan qRTPCRs was GAPDH, expect for experiment D‑0011400‑01‑05 (GAPDH) where the reference gene was ACTB (ß‑Actin). Dead cells (%) cell knockdown: percentage of missing cells using scrambled-RNAi or mock-transfection controls as the references for the number of cells.

| Samples or lncRNA Genbank accession number | siRNA | TaqMan PCR knockdown (%) | | MTT  Dead cells (%) | Trypan blue  Dead cells (%) | Dead% ± SD | Cell death efficiency Ranking |
| --- | --- | --- | --- | --- | --- | --- | --- |
|  |  | Scamble | Mock |  |  |  |  |
| Mock | ‑ | ‑ | 1.0 | ‑12 | ‑ |  |  |
| Apoptosis Tox | ‑ | ‑ | ‑ | 78 | 57 |  |  |
|  | D‑001210‑01‑05 Scramble | 0 | ‑ | 0 | 16 |  |  |
|  | D‑0011400‑01‑05 GAPDH (*) | 80 | 80 | ‑18 | 13 |  |  |
| AL833160 | **GGGCTTTAATTCTGCGAGA** | 82 | 62 | 56 | 41 | 54 ± 10 (**) | 1 |
|  | **CCCCAATCCACTCGAGAGA** | 64 | 24 | 64 | 55 |  |  |
| AF086466 | **GAACAATGAGCATAGCAAA** | 33 | 25 | 42 | 43 | 46 ± 8 (**) | 2 |
|  | **GGAATGTCCTGGGAAGCAT** | 4 (↑) | 17 (↑) | 58 | 40 |  |  |
| BC016787 | **GGAAATATGCACCCTGAGA** | 72 | 68 | 60 | 39 | 43 ± 11 (**) | 3 |
|  | **GTACAAAATAGACAGACAA** | 44 | 57 | 40 | 34 |  |  |
| BC039678 | **CATCTCAGCAATCGGGATA** | 63 | 57 | 35 | 29 | 37 ± 9 (**) | 4 |
|  | **GGAATGCAACCTTTCGACA** | 86 | 84 | 50 | 35 |  |  |
| hTF30525 | **CATCTGAACCTGACAAATA** | 14 | 11 | 28 | 40 | 36 ± 6 (***) | 5 |
|  | **TCAUAACAGCTTTGAGGTT** | 0 | 0 | 38 | 39 |  |  |
| BC038366 | **GAAGGAAGCAGGACGCAAT** | 84 | 70 | 52 | 35 | 32 ± 16 (*) | 6 |
|  | **CGACGTCACTTGCGCGTTT** | 85 | 71 | 15 | 27 |  |  |
| CR592608 | **CTACAGTACCTTAGGGAAA** | 49 | 41 | 37 | 29 | 27 ± 8 (**) | 7 |
|  | **AGAGAAGGATGAAGTGAAA** | 44 | 35 | 20 | 21 |  |  |
| AK025743 | **ACACATGCACTTACGTAAA** | 60 | 28 | 13 | 22 | 26 ± 11 (*) | 8 |
|  | **CAACAAAACCCACGGATGA** | 77 | 57 | 38 | 32 |  |  |
| AF251187 | **CATCCAACCTGTAGAGTAA** | 16 | 53 | 30 | 55 | 25 ± 25 | 9 |
|  | **AATAAGAGAACACTTCGTA** | 17 | 54 | ‑4 | 19 |  |  |
| BC038580 | **CTGCAGAGGCAATAAACAA** | 0 | 30 | 28 | 23 | 22 ± 7 (**) | 10 |
|  | **GCAGGTAGGCAGTGAGGAT** | 0 | 29 | 12 | 24 |  |  |
| CR593775 | **CACAAAGCTCAGACCGCCA** | 82 | 84 | 15 | 28 | 21 ± 7 (**) | 11 |
|  | **GCCAACTGGTACATCTAAT** | 89 | 89 | 15 | 24 |  |  |
| X15675 | **CTACCTAGTTGATGTCTTA** | 74 | 23 | 13 | 19 | 17 ± 4 (**) | 12 |
|  | **CCTAGTTGATGTCTTATAT** | 78 | 35 | 15 | 21 |  |  |
| BC040572 | **GGTGATAGCTAGAAGGTAA** | 0 | 81 | 10 | 20 | 16 ± 4 (**) | 13 |
|  | **TGAAAGAGAATGTGGTTAA** | 76 | 95 | 17 | 18 |  |  |
| AK127565 | **AGTTGAAGGTGGCCCCAAA** | 41 | 55 | 0 | 27 | 12 ± 12 | 14 |
|  | **CCAGGACTTCTGCAGGAGA** | 63 (↑) | 24 (↑) | 5 | 17 |  |  |
| CR612213 | **CAGCAGGAAGGGAGAACAA** | 27 | 61 | 17 | 23 | 12 ± 16 | 15 |
|  | **CCAGGATGGAGGAGGAGGA** | 42 (↑) | 25 | ‑11 | 20 |  |  |

*** pvalue <0.001, ** pvalue < 0.01, * pvalue <0.05 (^↑^): Indicate upregulation instead of knockdown (both siRNAs against AK057709 yielded upregulation, and hence that lncRNA is not reported)

BC041455 is an estrogen-repressed lncRNA. It was therefore not in the original siRNA screen of the 15 lncRNAs shown above, but was done later in order to produce the data for Figures 4F, 4G, and 4H.

| lncRNA | siRNA | TaqMan PCR knockdown (%) | | MTT  Dead cells (%) |
| --- | --- | --- | --- | --- |
|  |  | Scramble | Mock |  |
| BC041455 | **GGGTGAGACCTGATGGGAATT**  **GGCCTGGGGTGATGGAATATT** | 67.43 | 60.99 | -40 |
