# Supplementary material for: Primate-specific oestrogen-responsive long non-coding RNAs regulate proliferation and viability of human breast cancer cells
Source: Open Biol. 2016 Dec 21;6(12):150262. doi: 10.1098/rsob.150262 (PMC5204119; doi:10.1098/rsob.150262)
Supplement: Primate-specific oestrogen-responsive long non-coding RNAs regulate proliferation and viability of human breast cancer cells, Lipovich et al. Supplementary Table 4 [file rsob150262supp4.docx]

**Supplementary Table 4: TaqMan combos (probe/primers) used for qRTPCR.**

| LifeTech catalog number | lncRNA Accession Number |
| --- | --- |
| Custom | BC016787 |
| Custom | HTF30525 |
| Custom | AF086466 |
| Custom | BC036599 |
| Custom | AK057709 |
| Custom | AF251187 |
| Custom | BC039678 |
| Custom | BC040572 |
| Custom | CR593775 |
| Custom | AK127565 |
| Hs03866108_s1 | AK024898 |
| Hs03668510_s1 | AK025743 |
| Hs03680777_m1 | AK09603 |
| Hs03868638_s1 | AK096780 |
| Hs03886156_s1 | AK123408 |
| Hs03842484_s1 | AL832444 |
| Hs03673766_s1 | AL833160 |
| Hs03674088_s1 | BC038366 |
| Hs03918292_s1 | BC038557 |
| Hs03850343_s1 | CR592608 |
| Hs03921574_s1 | X15675 |
| Hs03299152_m1 | LIT3339 |
| Hs03861227_s1 | CR610499 |
| Hs00288663_m1 | CR612213 |
| Hs00862171_m1 | BC041455 |
| Hs01377128_m1 | BC038580 |
