# Supplementary material for: Primate-specific oestrogen-responsive long non-coding RNAs regulate proliferation and viability of human breast cancer cells
Source: Open Biol. 2016 Dec 21;6(12):150262. doi: 10.1098/rsob.150262 (PMC5204119; doi:10.1098/rsob.150262)
Supplement: Supplementary Figure 7 [file rsob150262supp7.pdf]

Supplementary Fig. 7 (A)

Stellaris fluorescent *in situ* hybridization (FISH) demonstrating cytoplasmic localization of BC041455 lncRNA in MCF7 cells

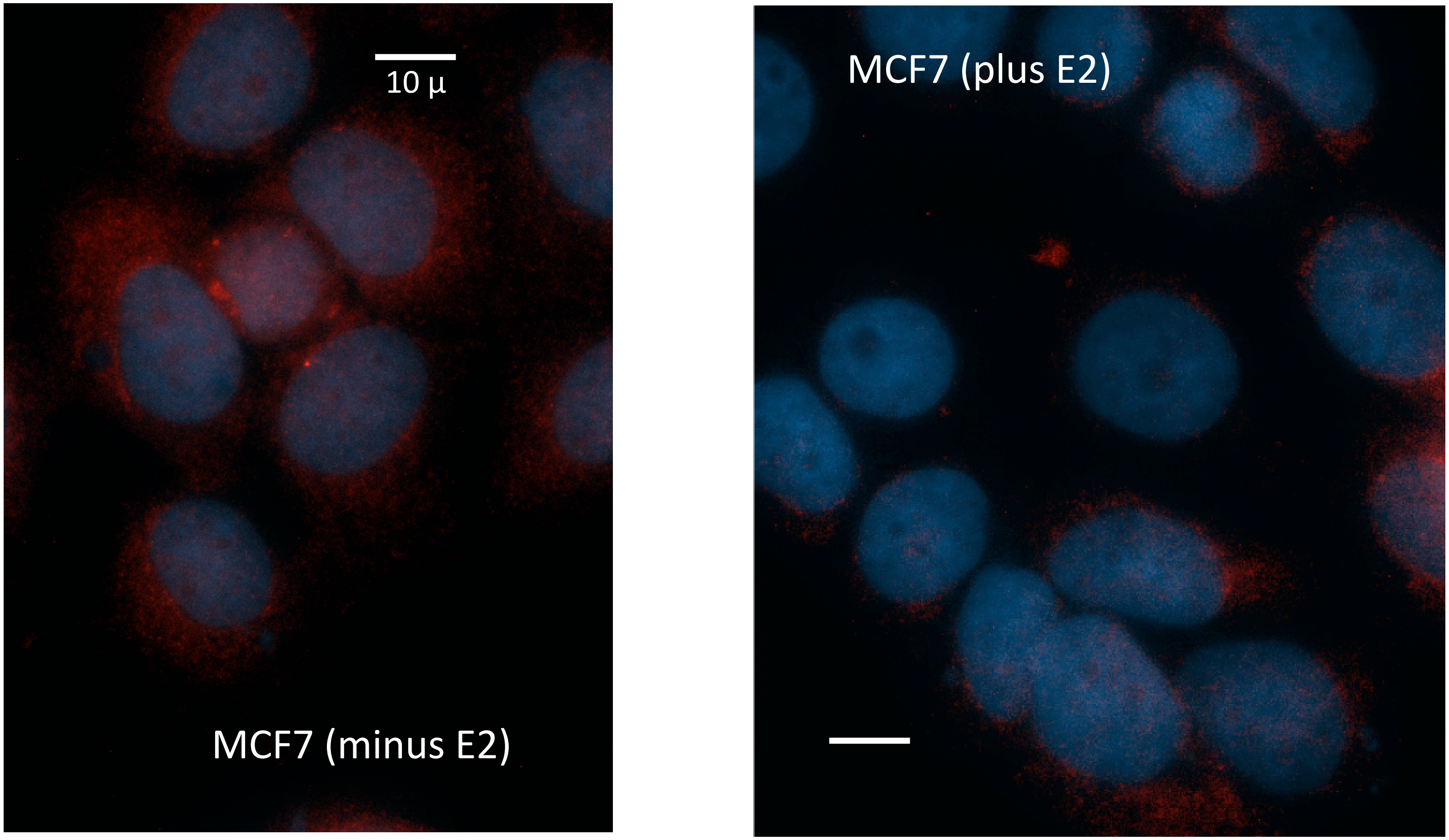

Supplementary Fig. 7 (B)

Stellaris fluorescent *in situ* hybridization (FISH) demonstrating cytoplasmic localization of BC041455 lncRNA in T47D cells

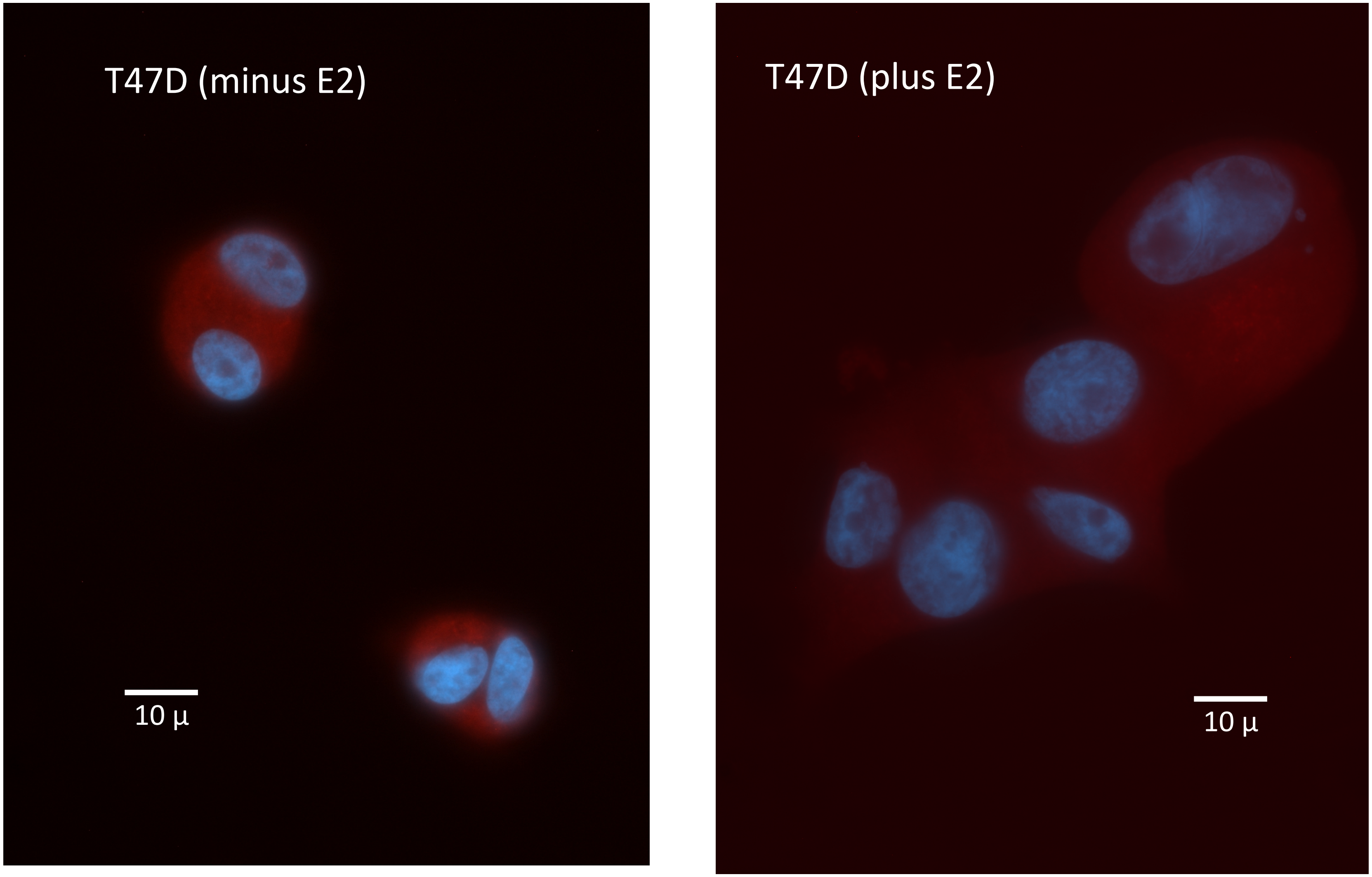

Supplementary Fig. 7 (C)

Stellaris fluorescent *in situ* hybridization (FISH) demonstrating cytoplasmic localization of BC041455 lncRNA in T47D cells

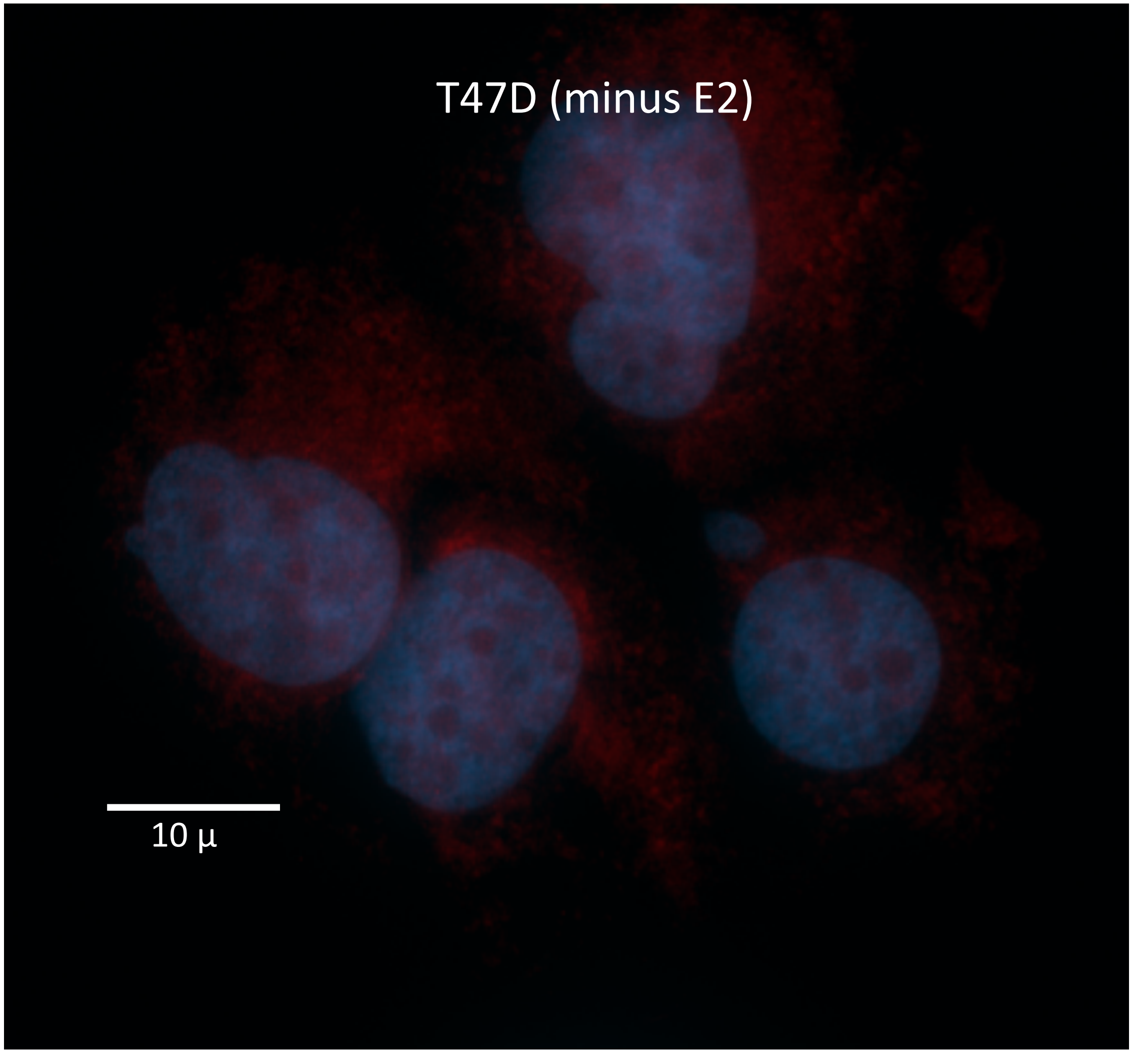

Supplementary Fig. 7 (D)

Stellaris fluorescent *in situ* hybridization (FISH) demonstrating cytoplasmic localization of BC041455 lncRNA in MDA-MB-231 cells

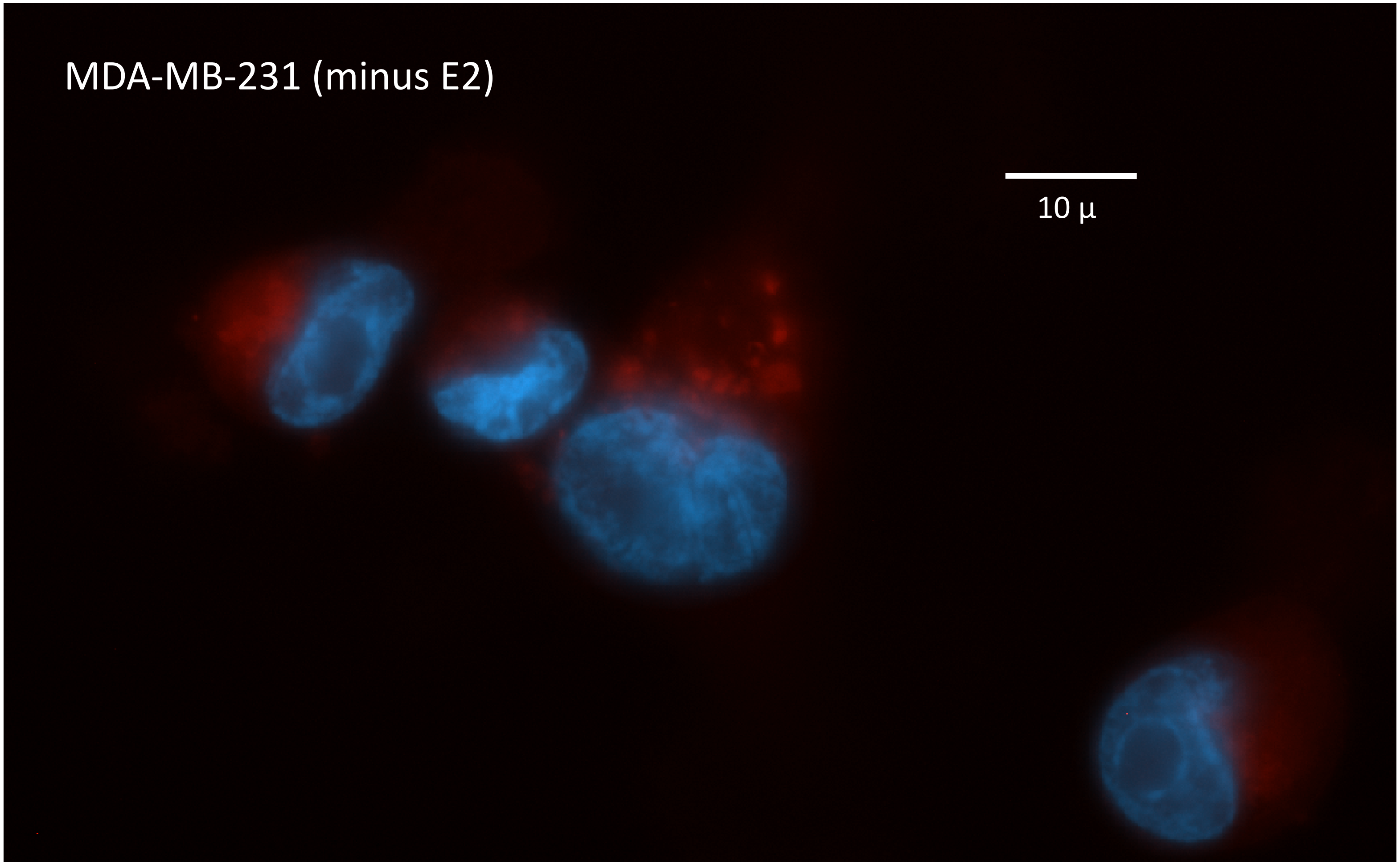

Supplementary Fig. 7 (E)

Stellaris fluorescent *in situ* hybridization (FISH) comparing cytoplasmic BC041455 lncRNA in MCF7 cells to GM03814 fibroblasts

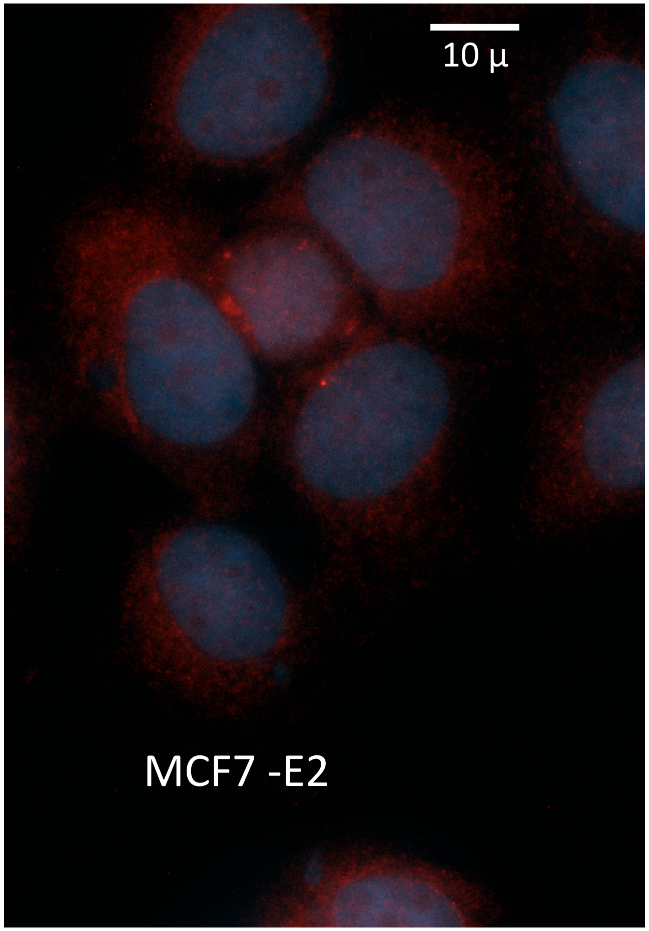

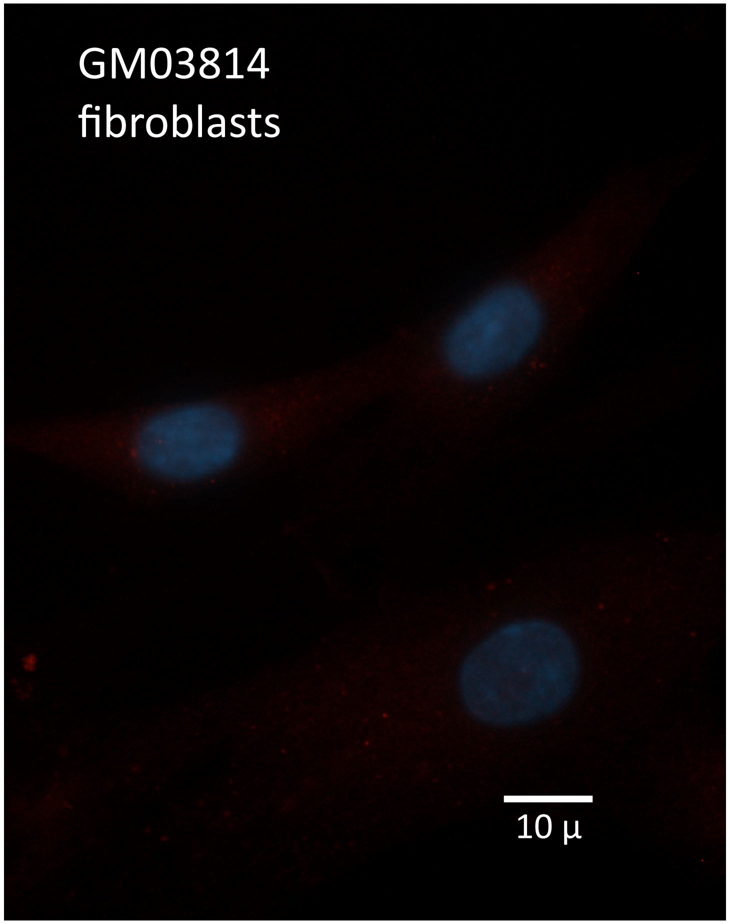

Supplementary Fig. 7 (F)

Sample screenshot of the entire Zen software screen analyzing z-stack and fluorescence acquired on the Zeiss AxioObserver microscope

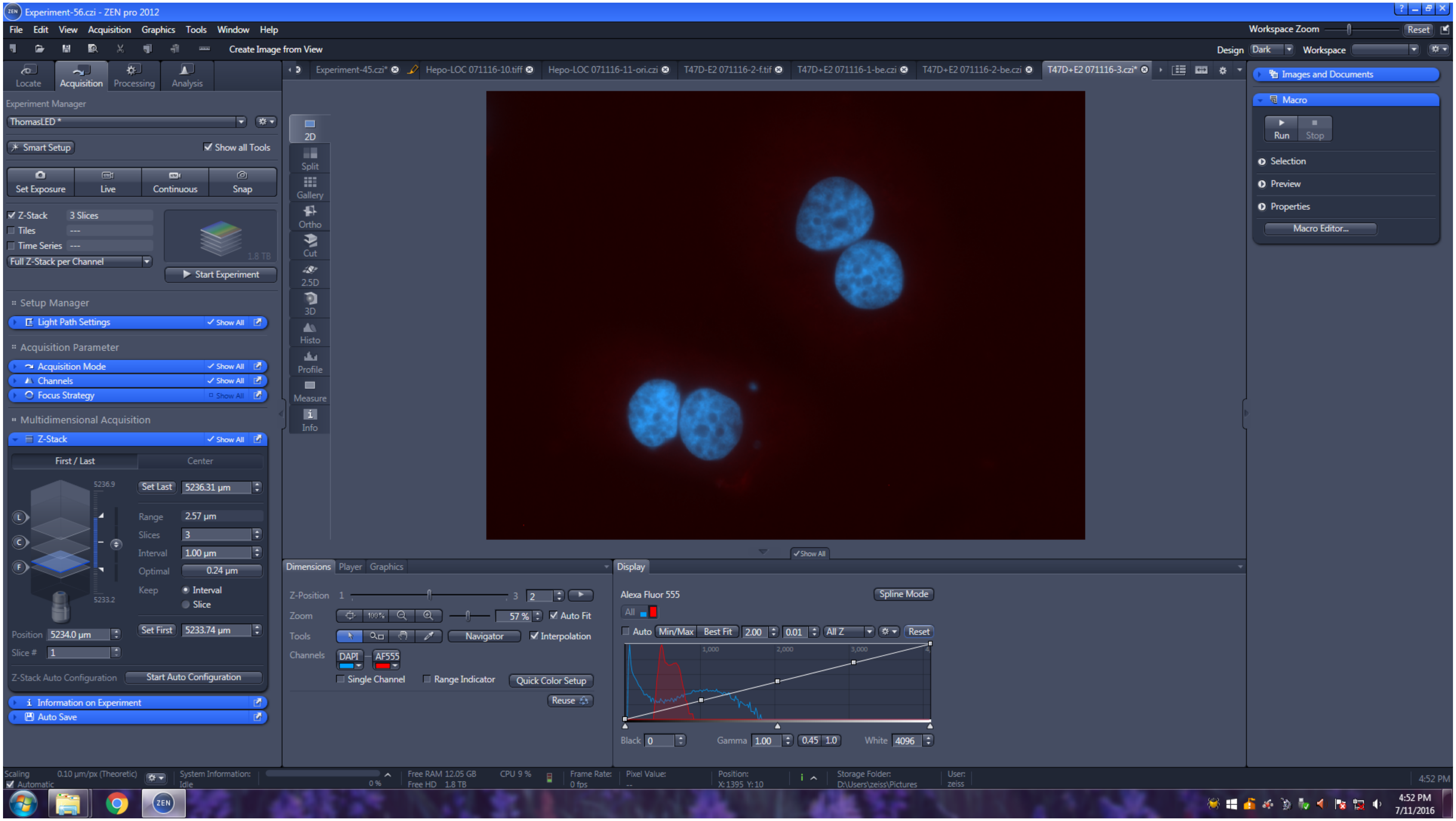

Figure Legend for Suppl Fig 7:

For T-47D and MCF-7 (Panels A and B), cells were plated onto collagen-coated 25 mm diameter glass coverslips (Fisher Scientific 12-545-86; 0.13 to 0.17 mm thick) in estrogen-free medium (i.e., 10% charcoal-dextran-stripped FBS with pheno-red free basal media). Cells were allowed to grow overnight before stimulation with 10 nM β-estradiol or vehicle (ethanol), and then rinsed 1X with PBS before fixation in 4% paraformaldehyde (made up in 1X PBS) for 20 min, followed by multiple washes in 1X PBS. Coverslips were stored at -20°C in 70% ethanol until the hybridization step. A positive control was the estrogen-receptor negative human breast cancer cell line MDA-MB-231 (Panel C) grown in normal media (10% FBS with pheno red). A negative control was provided using the normal adult female human fibroblast line GM03814 (Coriell Institute for Medical Research). In **Panel A**, we show the BC041455 FISH result on MCF-7 cells ± E2 restimulation; the punctate staining is similar to Type V described by Cabili et al. (2015) over the cytoplasm, with little or no signal over the DAPI (blue)-stained nuclei. Treatment of MCF-7 cells with E2 (Panel A, right) strongly reduces the Quasar 570 signal, consistent with TaqMan qRT-PCR results which reveal that E2 treatment of MCF7 cells strongly suppresses the steady-state level of BC041455 lncRNA (by ~2 C_T values; data not shown). The overall Quasar 570 signal in T-47D cells (**Panel B**) is stronger than the signal seen in MCF-7 cells (Panel A), with less E2 suppression, consistent with our TaqMan measurements, which show that the steady-state level of BC041455 lncRNA in T-47D cells averages 3-4 C_T steps less than MCF7 cells. The red fluorescence in T47D cells is very bright compared to MCF7 cells (Panel B), so in **Panel C**, we increase maginification and turned down the gain on the red channel to visualize the distinct small puncta. In **Panel D**, we show the BC041455 signal in the human breast cancer line MDA-MB-231 which is ER-negative; the shape of the nuclei is different here than the ER-positive cell lines, but the Quasar 570 signal is nearly as strong as seen in T-47D cells. In **Panel E**, we compare side-by-side MCF-7 minus E2 cells to normal female human fibroblasts (GM03814), demonstrating that the fibroblast signal is virtually absent compared to the breast cancer cells. Finally, TaqMan RT-PCR measurements of BC041455 lncRNA in the normal human fibroblast GM03814 is nearly undetectable (C_T >36), about 11 C_T steps later in real-time PCR than T-47D, or 2^{11} (=2048 times less signal). Thus, the cytoplasmic Stellaris FISH signals in the breast cancer cell lines are chiefly cytoplasmic, and quantitatively in line with values obtained by TaqMan qRT-PCR. All white bars designate 10 μ. Acquired data was kept in .czi files created by ZEN software 2012 “Blue” (see **Panel F**). In the bottom of Panel F, we demonstrate the use of the slider bar to separate the “noise” red peak (left) from the “signal” red peak (right), guaranteeing that our red signal is indeed coming from Quasar 570 and not autofluorescence.
