# Supplementary material for: Primate-specific oestrogen-responsive long non-coding RNAs regulate proliferation and viability of human breast cancer cells
Source: Open Biol. 2016 Dec 21;6(12):150262. doi: 10.1098/rsob.150262 (PMC5204119; doi:10.1098/rsob.150262)

T47D Scramble (-E2)

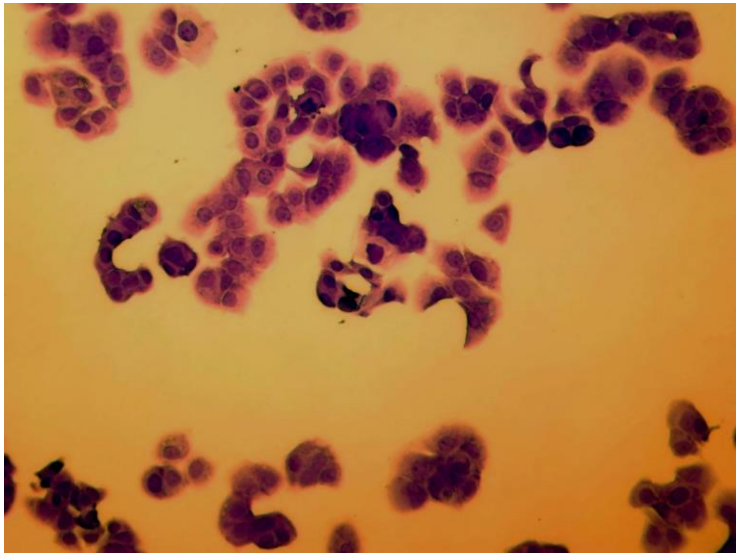

T47D siRNA BC041455 (-E2)

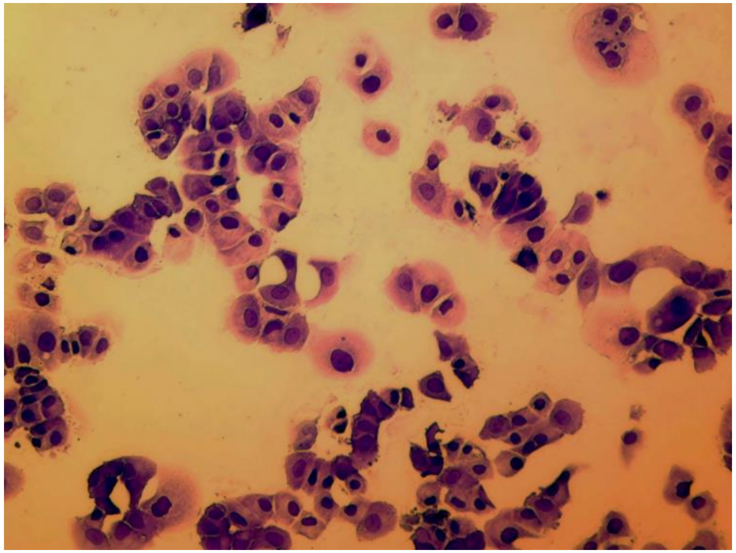

MCF7 Scramble (-E2)

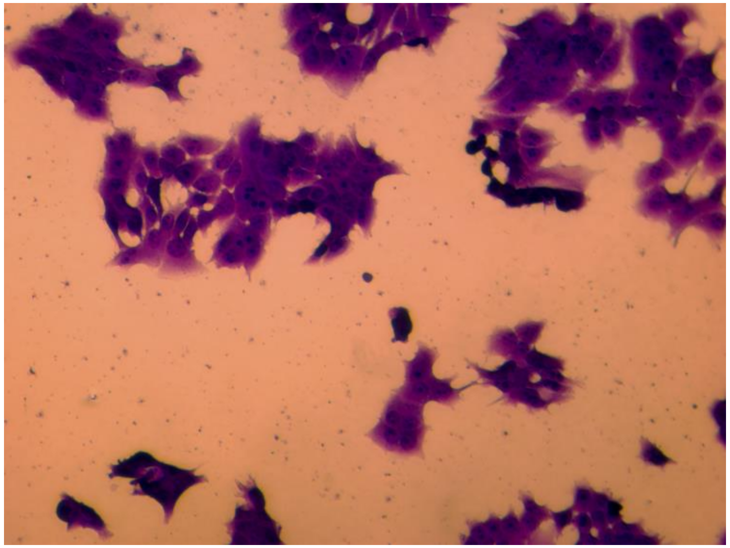

MCF7 siRNA BC041455 (-E2)

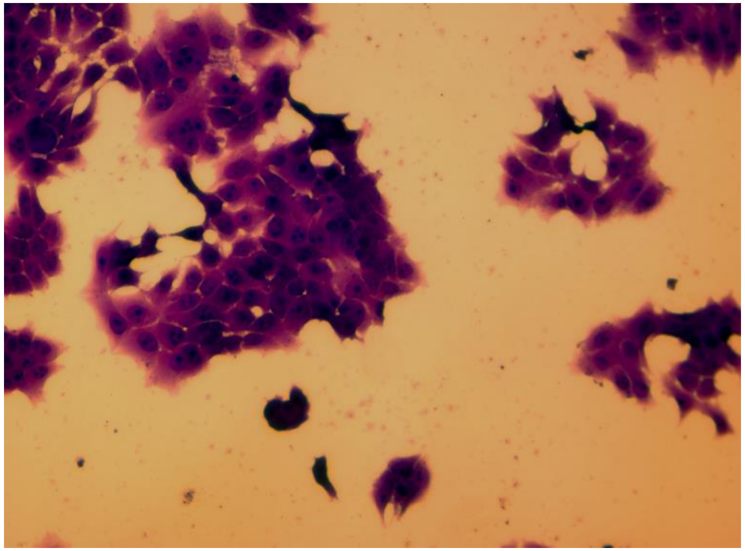

T47D Empty Vector (-E2)

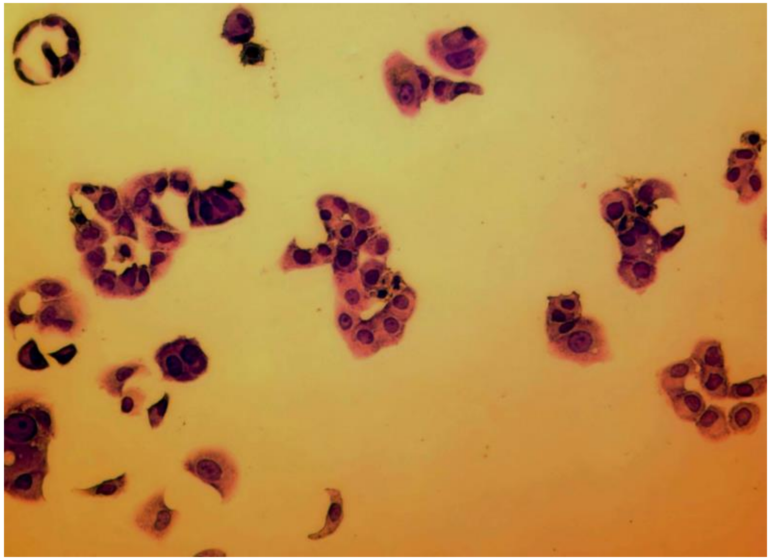

T47D CR593775 Over Expression (-E2)

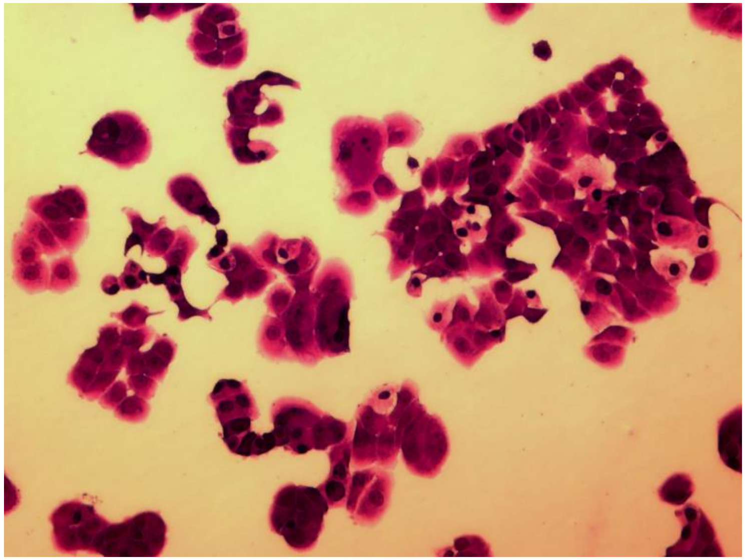

MCF7 Empty Vector (-E2)

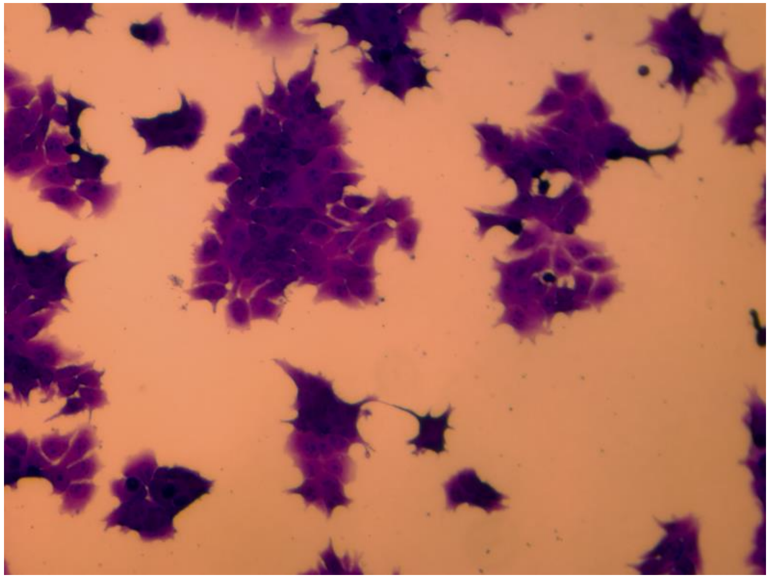

MCF7 CR593775 Over Expression (-E2)

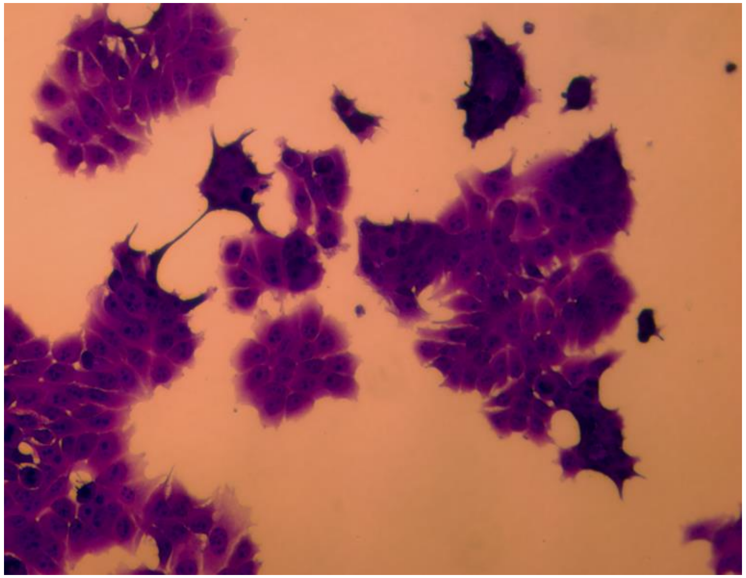

Supplement: Supplementary Figure 8 [file rsob150262supp8.pdf]
