# Supplementary material for: Primate-specific oestrogen-responsive long non-coding RNAs regulate proliferation and viability of human breast cancer cells
Source: Open Biol. 2016 Dec 21;6(12):150262. doi: 10.1098/rsob.150262 (PMC5204119; doi:10.1098/rsob.150262)
Supplement: Supplementary Figure 9 [file rsob150262supp9.pptx]

## Slide 1
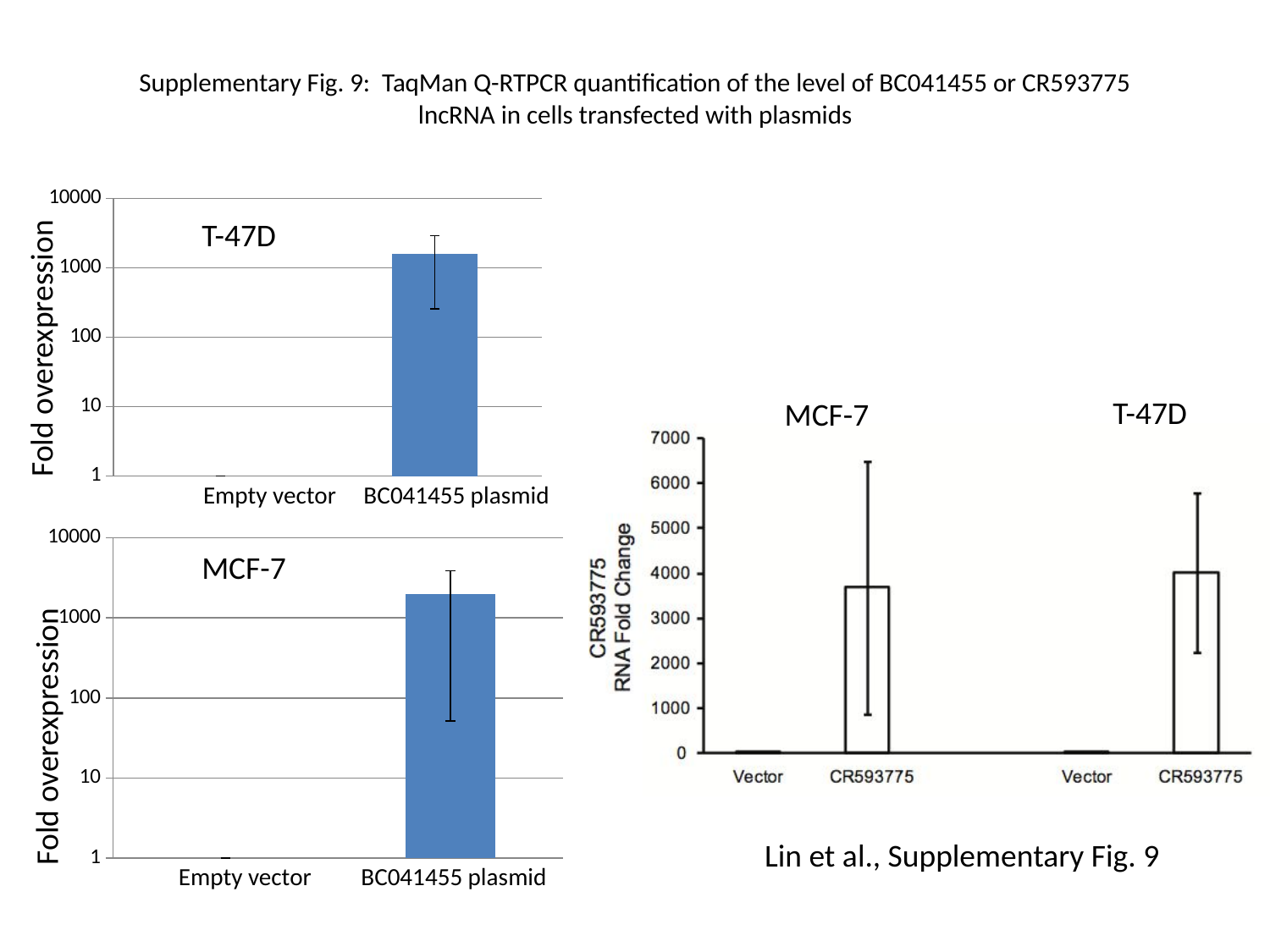

# Supplementary Fig. 9: TaqMan Q-RTPCR quantification of the level of BC041455 or CR593775 lncRNA in cells transfected with plasmids
### Chart
| Category | |
|---|---|
| T47d sport6 ctr | 1.0 |
| T47D BC041455 | 1571.143580976254 |T-47D
Fold overexpression
T-47D
MCF-7
Empty vector BC041455 plasmid
### Chart
| Category | |
|---|---|
| MCF7 sport6 ctr | 1.0 |
| MCF7 BC041455 | 1961.822499974569 |MCF-7
Fold overexpression
Lin et al., Supplementary Fig. 9
Empty vector BC041455 plasmid
