# Supplementary material for: Primate-specific oestrogen-responsive long non-coding RNAs regulate proliferation and viability of human breast cancer cells
Source: Open Biol. 2016 Dec 21;6(12):150262. doi: 10.1098/rsob.150262 (PMC5204119; doi:10.1098/rsob.150262)
Supplement: Supplementary Figure 10 [file rsob150262supp10.ppt]

## Slide 1
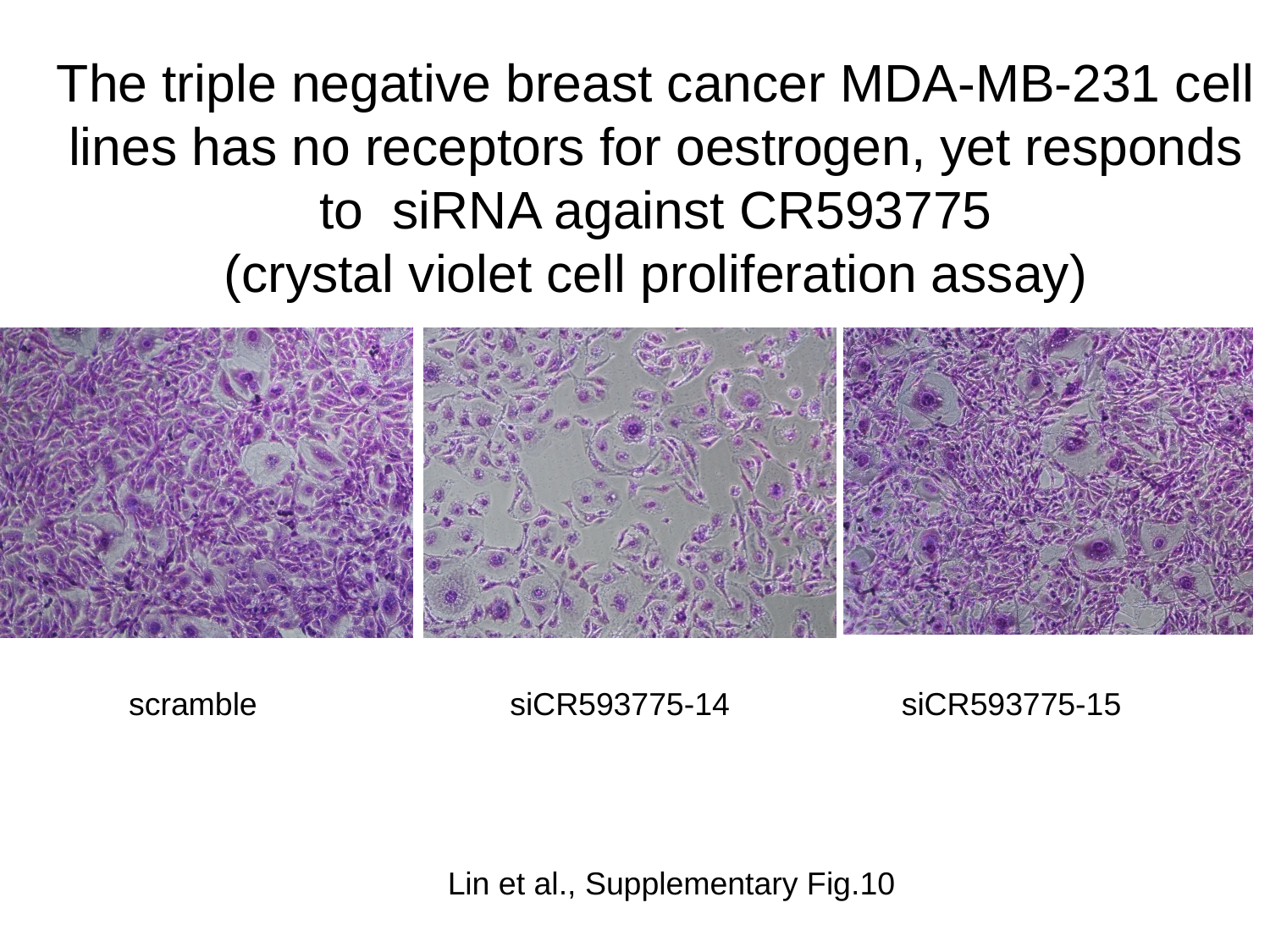

The triple negative breast cancer MDA-MB-231 cell lines has no receptors for oestrogen, yet responds to siRNA against CR593775
(crystal violet cell proliferation assay)
scramble
siCR593775-14
siCR593775-15
Lin et al., Supplementary Fig.10
