# Supplementary material for: Primate-specific oestrogen-responsive long non-coding RNAs regulate proliferation and viability of human breast cancer cells
Source: Open Biol. 2016 Dec 21;6(12):150262. doi: 10.1098/rsob.150262 (PMC5204119; doi:10.1098/rsob.150262)
Supplement: Supplementary Figure 11 [file rsob150262supp11.pptx]

## Slide 1
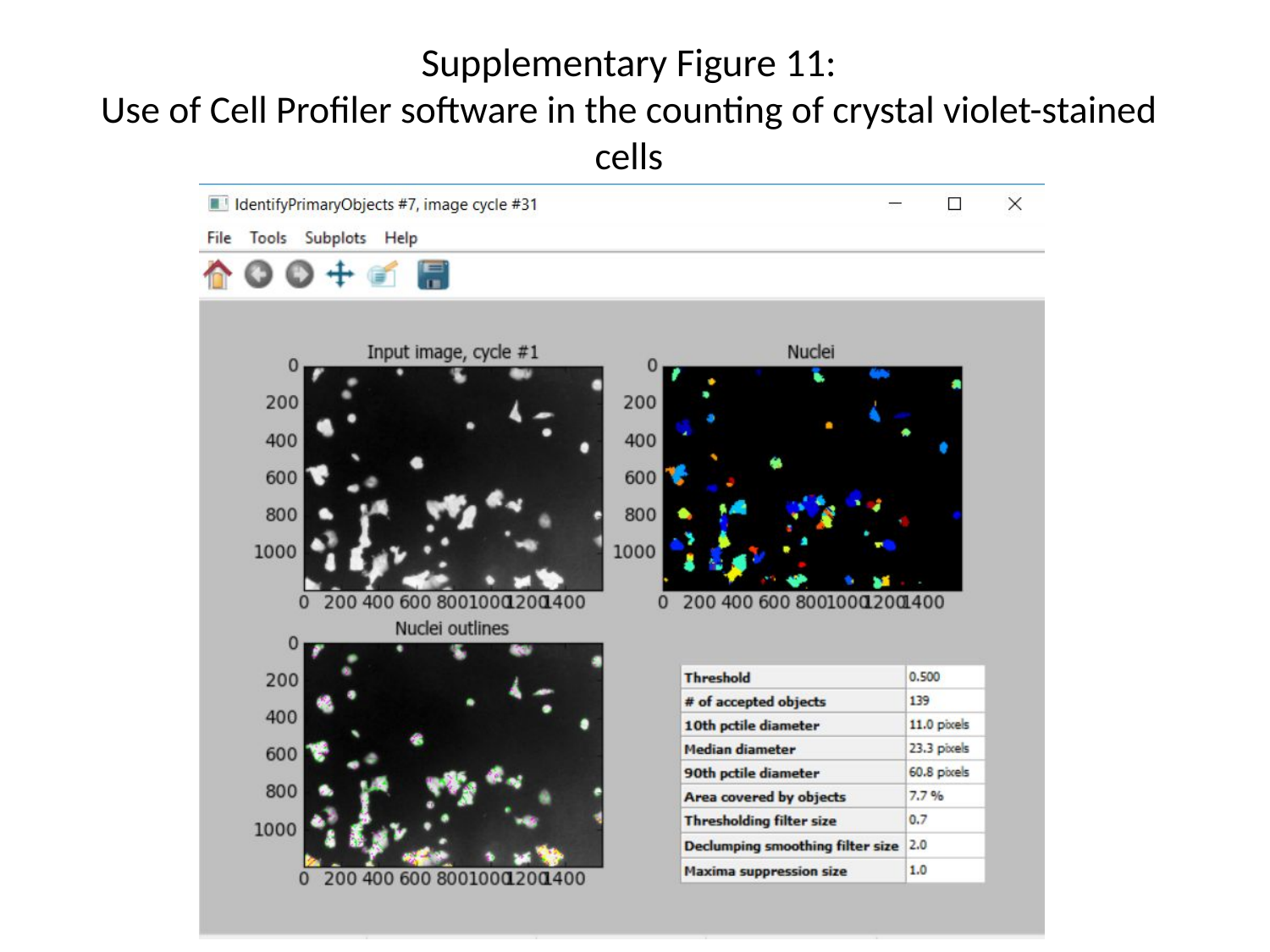

# Supplementary Figure 11: Use of Cell Profiler software in the counting of crystal violet-stained cells
