# Supplementary material for: Primate-specific oestrogen-responsive long non-coding RNAs regulate proliferation and viability of human breast cancer cells
Source: Open Biol. 2016 Dec 21;6(12):150262. doi: 10.1098/rsob.150262 (PMC5204119; doi:10.1098/rsob.150262)
Supplement: ENCODE Gingeras [file rsob150262supp12.docx]

**New supplementary document**

**Panel A: ENCODE Gingeras stranded CSHL Long RNAseq Track of BC041455**


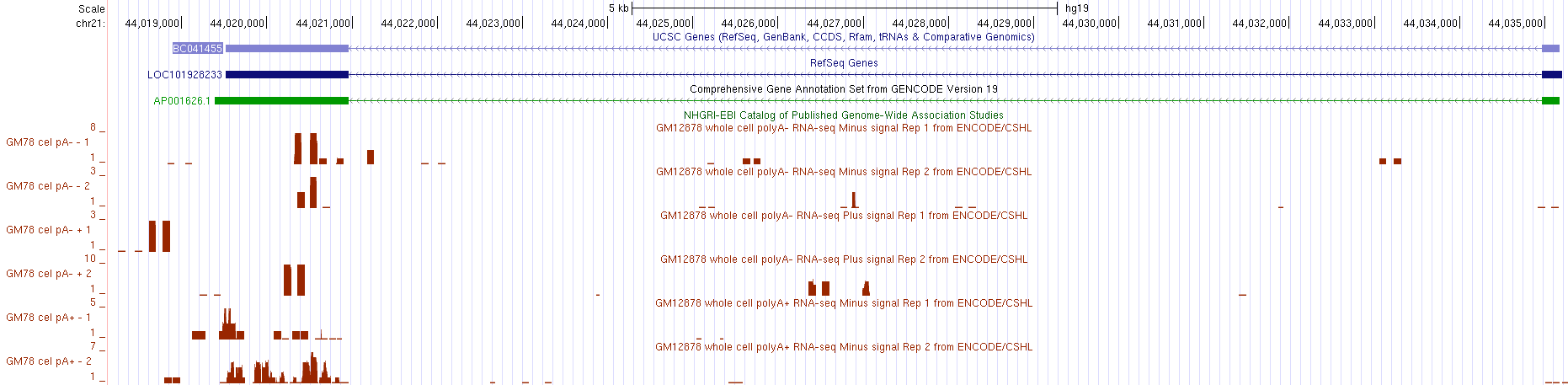


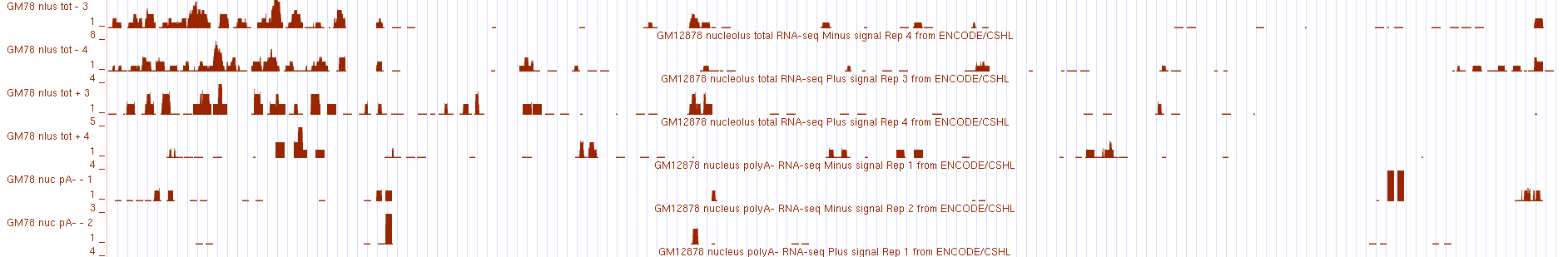


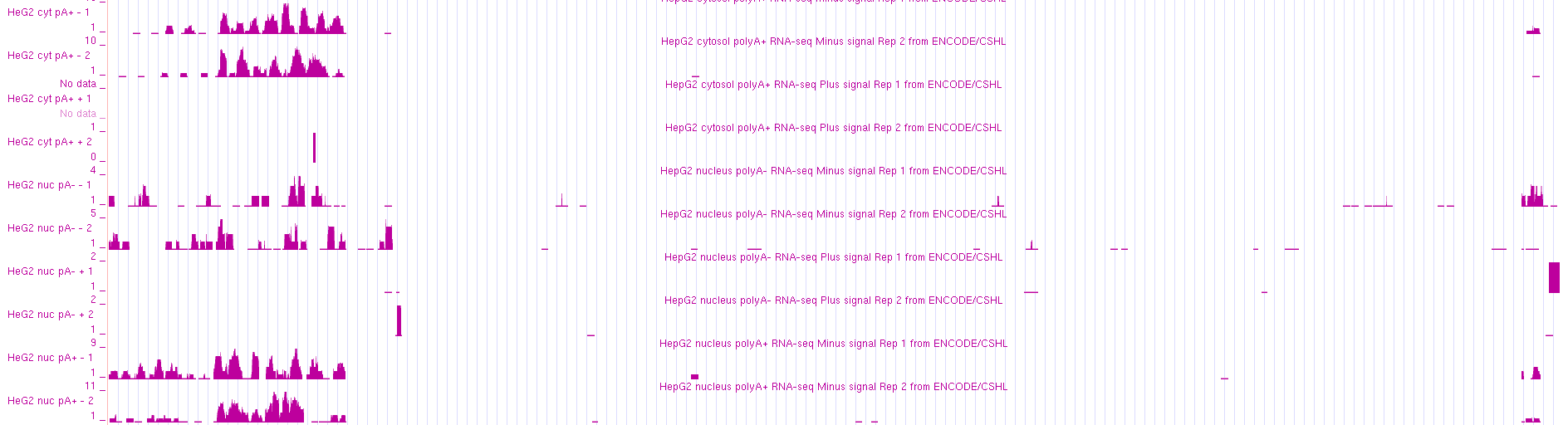


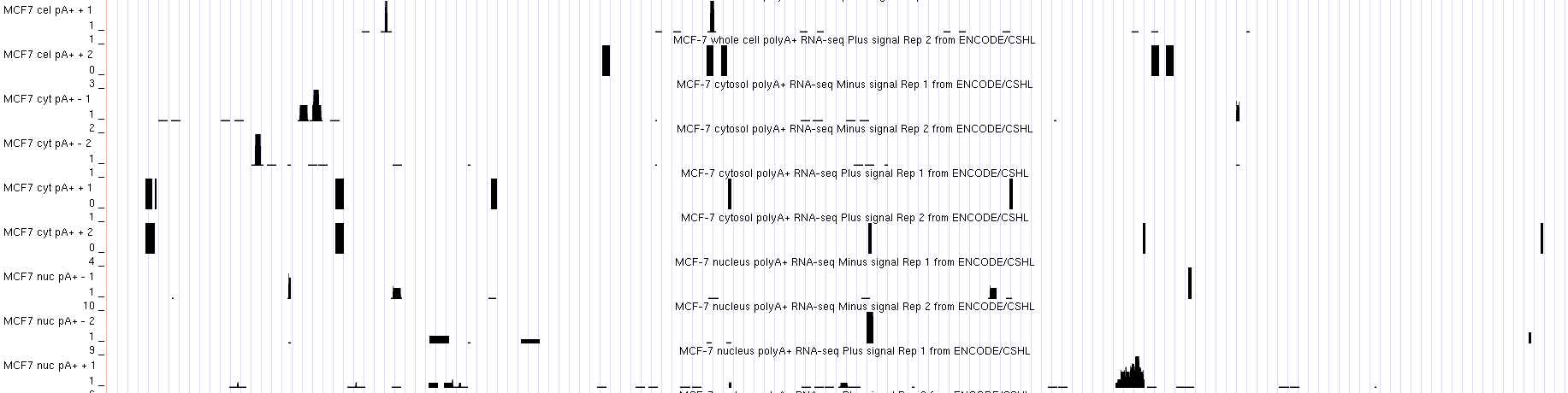


**Panel B: ENCODE Gingeras Stranded CSHL Long RNAseq Tracks in CR593775 (LOC105447648)**


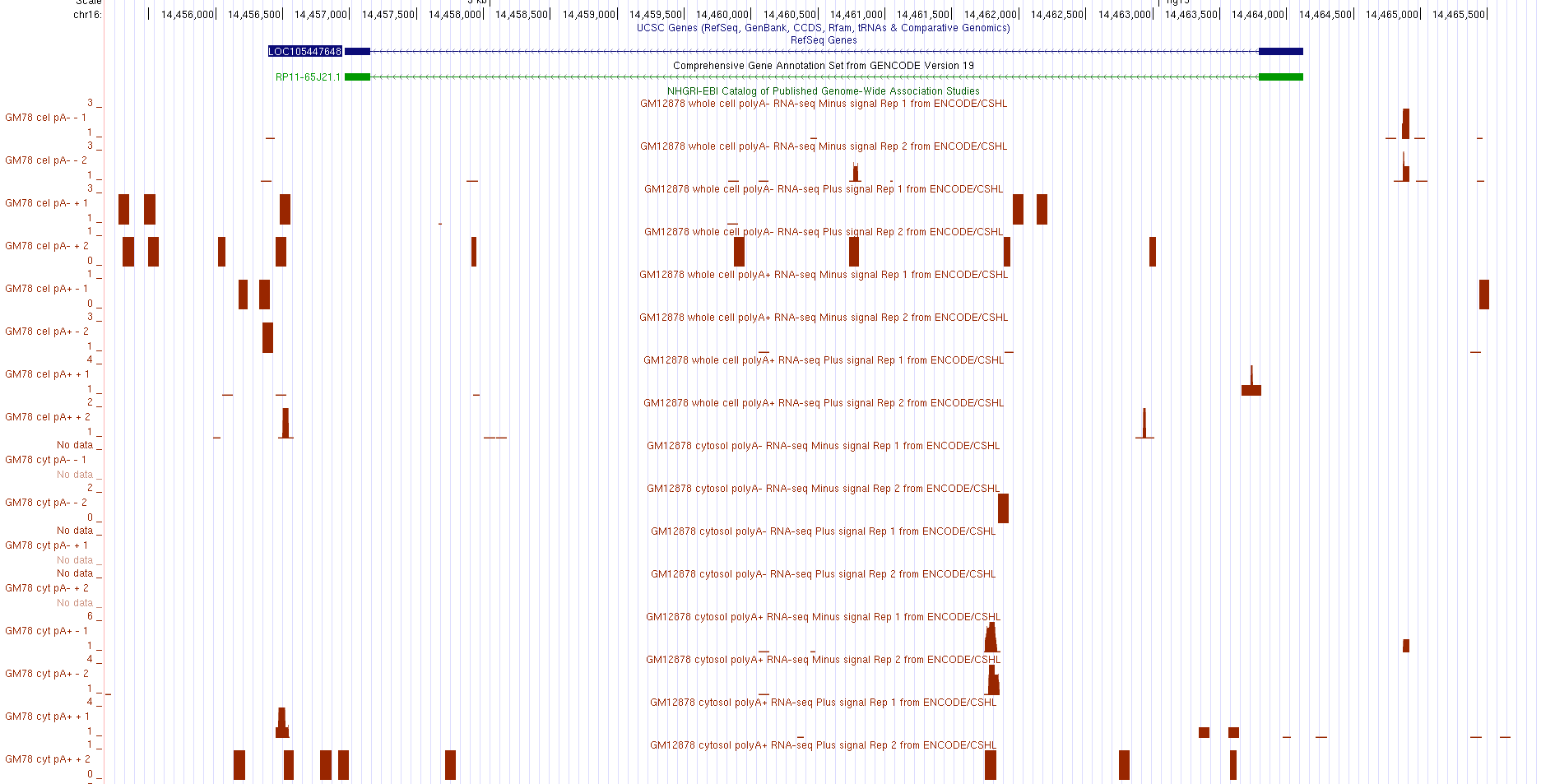


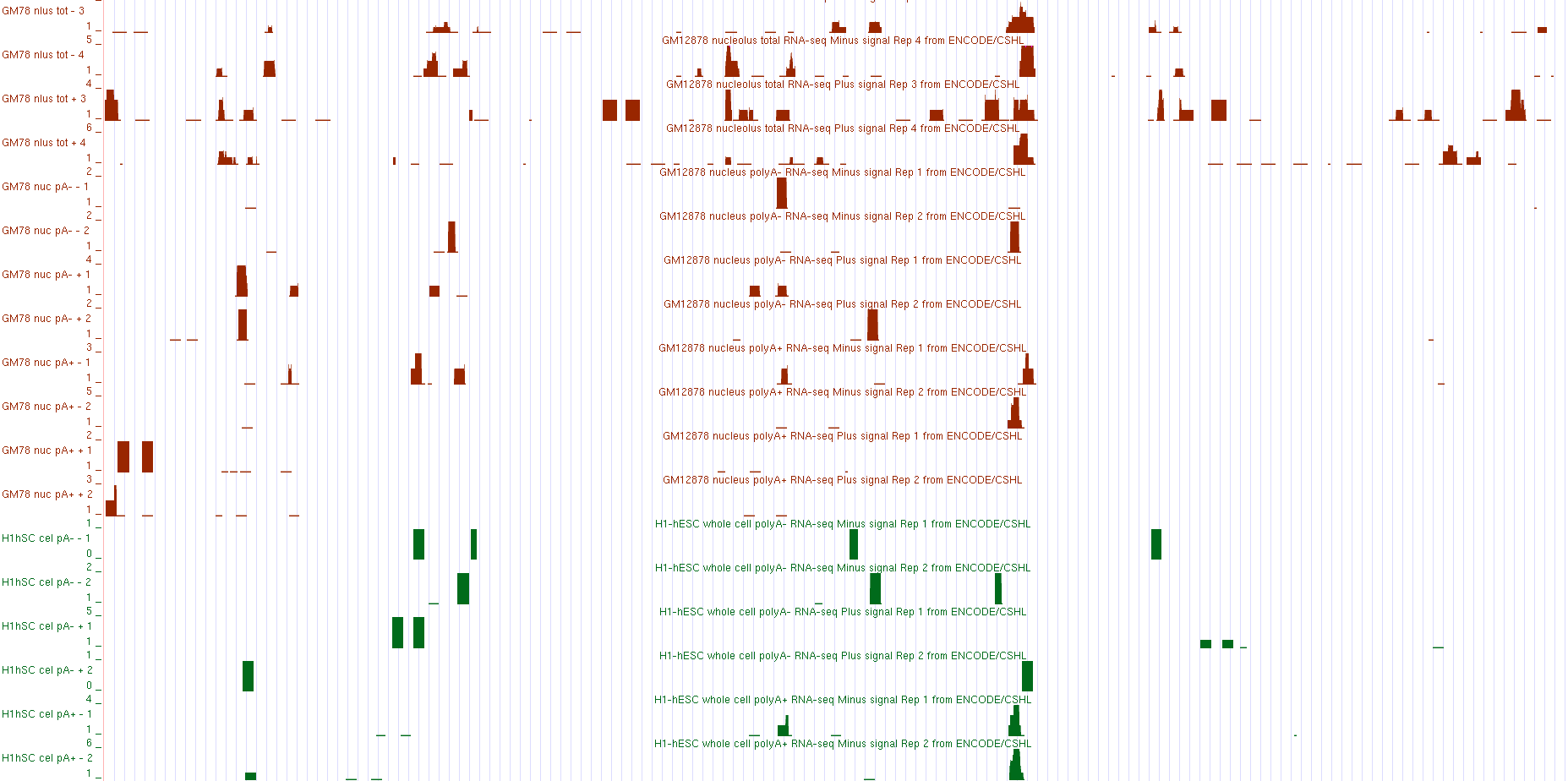


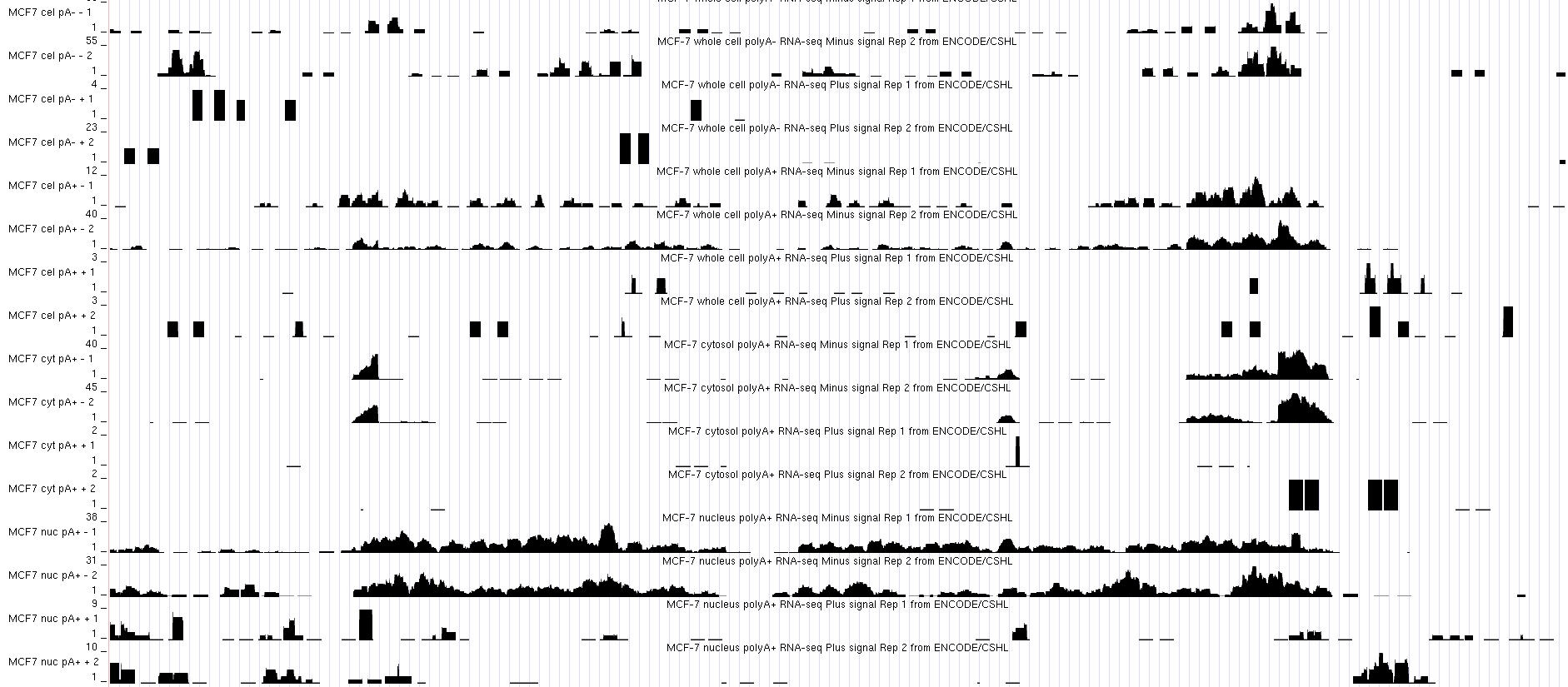


**Figure Legend:**

Panel A: Red boxes on the figure indicate the position of the two exons in BC041455 (LOC101928233). The red arrow denotes the direction of transcription.

Panel B: Red boxes on the figure indicate the position of the two exons in LOC105447648 (CR593775). The red arrow denotes the direction of the transcription. The blue box on the left margin of Panel B highlights the RNA-seq tracks for the cell line MCF-7. The orange boxes highlights the RNA-seq tracks in the two exons; the blue arrows point to the splice donor and splice acceptor features that are very sharp at the splice boundary.

Methods of how to display the ENCODE Gingeras Standard CSHL Long RNA-seq Tracks

1. Go to genome.ucsc.edu
2. Click on genome browser and type in the search box, in this case we choose BC041455. Select the BC041455 below and click go.
3. To display the RNA-seq data, select the setting below in the categories “Expression “and select ENC RNA seq.
4. Once we select the ENC RNA-seq, the screen will display ENCODE RNA-seq tracks setting. Select hide on Caltech, GIS, HAIB, and SYDH. Select full on CSHL Long RNA-seq.
5. Click on CSHL Long RNA-seq. Set the maximum display to full mode. Hide the contigs. Set the plus and minus signal to full. Then click on the plus and minus signal, set the track height to 36 and data view scaling to auto-scale to data view.
6. Select subtracks by localization and cell line by clicking on plus and minus sign.
7. Once the selection made, submit.
8. The genome browser will display the ENCODE Gingeras Standard CSHL RNA-seq Tracks.
